# In silico design of refined ferritin-SARS-CoV-2 glyco-RBD nanoparticle vaccine

**DOI:** 10.3389/fmolb.2022.976490

**Published:** 2022-09-06

**Authors:** Seyedeh Zeinab Masoomi Nomandan, Maryam Azimzadeh Irani, Seyed Masoud Hosseini

**Affiliations:** Faculty of Life Sciences and Biotechnology, Shahid Beheshti University, Tehran, Iran

**Keywords:** *in silico* vaccine design, ferritin nanoparticle vaccine, SARS-CoV-2 RBD, molecular modeling, molecular dynamics simulation

## Abstract

With the onset of Coronavirus disease 2019 (COVID-19) pandemic, all attention was drawn to finding solutions to cure the coronavirus disease. Among all vaccination strategies, the nanoparticle vaccine has been shown to stimulate the immune system and provide optimal immunity to the virus in a single dose. Ferritin is a reliable self-assembled nanoparticle platform for vaccine production that has already been used in experimental studies. Furthermore, glycosylation plays a crucial role in the design of antibodies and vaccines and is an essential element in developing effective subunit vaccines. In this computational study, ferritin nanoparticles and glycosylation, which are two unique facets of vaccine design, were used to model improved nanoparticle vaccines for the first time. In this regard, molecular modeling and molecular dynamics simulation were carried out to construct three atomistic models of the severe acute respiratory syndrome coronavirus 2 (SARS-CoV-2) receptor binding domain (RBD)-ferritin nanoparticle vaccine, including unglycosylated, glycosylated, and modified with additional O-glycans at the ferritin–RBD interface. It was shown that the ferritin–RBD complex becomes more stable when glycans are added to the ferritin–RBD interface and optimal performance of this nanoparticle can be achieved. If validated experimentally, these findings could improve the design of nanoparticles against all microbial infections.

## Introduction

Coronavirus disease 2019 (COVID-19), a highly contagious disease caused by severe acute respiratory syndrome coronavirus 2 (SARS-CoV-2), has infected more than 281 million people worldwide and caused more than 5.4 million deaths as of 29 December 2021 (World Health Organization., 2021). The COVID-19 pandemic caused by the SARS-CoV-2 virus causes enormous distress to millions of people worldwide and has long-term effects on all aspects of people’s lives.

Coronaviruses are large enveloped RNA-positive-stranded viruses, and the SARS-CoV-2 consists of a large RNA genome, four structural proteins, 16 nonstructural proteins, and nine to 11 accessory proteins (Michel et al., 2020; Redondo et al., 2021). The four structural proteins include the spike, envelope, membrane, and nucleocapsid proteins (Chan et al., 2020; Walls et al., 2020; Tao et al., 2021), of which the spike glycoprotein (S-protein) is of particular interest for coronavirus vaccine target (Bisht et al., 2004; Du et al., 2009; Fakih and Dewi, 2020; Yang et al., 2020).

Coronaviruses are a diverse group of viruses that includes the Middle East respiratory syndrome coronavirus (MERS-CoV) and severe acute respiratory syndrome coronavirus (SARS-CoV), infecting different animal species, and they can cause diseases of the upper respiratory tract, gastrointestinal tract, and central nervous system in humans and other animals (Andersen et al., 2020; Ganji et al., 2020; Wu et al., 2020; Zhou et al., 2020; Zhu et al., 2020; Zu et al., 2020; Yang et al., 2021). In 2002 and 2012, two highly pathogenic coronaviruses of animal origin, SARS-CoV and MERS-CoV, emerged and caused fatal respiratory illness (Corman et al., 2018; Cui et al., 2019; Chen Y. et al., 2020; Shereen et al., 2020). Hence, developing a safe and effective SARS-CoV-2 vaccine with antibody persistence and long-term memory to combat the deadly virus outbreak is a public health priority.

The spike protein with a functional polynucleotide furin cleavage site at its S1–S2 subdomain boundary plays an essential role in the infectivity of SARS-CoV-2 (Li et al., 2005; Song et al., 2018; Kar and Leszczynski, 2020; Walls et al., 2020; Wrapp et al., 2020). The active S protein is a trimer in which every monomer of it consists of a fusion peptide, two heptad repeats, an intracellular domain, an N-terminal domain, two subdomains, and a transmembrane region S-protein (Piplani et al., 2021). S-protein is a glycoprotein, and the attached glycans protect about 40% of the surface of the trimeric S protein, which serves as a camouflage for the humoral and cellular components of the host’s innate immune system (Rudd et al., 2001; Casalino et al., 2020; Choi et al., 2021).

The receptor binding domain of the spike protein binds to angiotensin-converting enzyme 2 (ACE2) ectodomain, creating significant immunogenicity among the other spike proteins, accounting for up to 90% of neutralizing antibodies (nAbs) obtained from convalescent serum (He et al., 2004, 2005; Lakshmanane et al., 2020). Importantly, patients with COVID-19 elicit a strong nAbs response to SARS-CoV-2 spikes, suggesting that this antigen is promising in protective vaccines (Robbiani et al., 2020).

Since the outbreak, several strategies have emerged to combat this deadly virus (Chen W.-H. et al., 2020; Dagotto et al., 2020; Haiou et al., 2022). However, the most promising strategy and long-term solution is the development of an effective vaccine. Various vaccine platforms have been developed, such as inactivated vaccines (Gao et al., 2020; Feng et al., 2021; Jara et al., 2021), DNA plasmid vaccines (Jingyou et al., 2020; Nishikawa et al., 2021), adenovirus-vectored vaccines (Buchbinder et al., 2020; Van Doremalen et al., 2020), RNA vaccines (Corbett et al., 2020; Jackson et al., 2020), protein subunit vaccines (Kanekiyo et al., 2013; Wang L. L. et al., 2017, 2020, 2021; Keech et al., 2020; Yang et al., 2020; Kalathiya et al., 2021; Powell et al., 2021) and virus-like particle vaccines (Dong et al., 2020; Krammer, 2020). Since the pandemic, RNA-based vaccines have attracted all the attention; nevertheless, subunit vaccines that use the spike protein to elicit a protective antibody response are attractive for accessible SARS-CoV-2 vaccines because of their safety, expandability in production, and ease of distribution to low-and middle-income countries (Wang et al., 2020).

Over the last few years, there have been tremendous advances in the field of protein-based nanomaterials, and among the various protein-based nanomaterials, protein nanocages are probably the most sophisticated in protein subunit vaccines (Liu et al., 2014; He et al., 2016; Votteler et al., 2016; Edwardson and Hilvert, 2019). Self-organization has inspired scientists in many disciplines, from a small number of subunits to a symmetric mono-distributed architecture. Protein cages can be considered polymer containers with various charge encapsulations and functions.

Protein subunit vaccines and genetically encoded nucleic acid vaccines are most effective for SARS-CoV-2 treatment (Amanat and Krammer, 2020). Although the immunogenicity of subunit vaccines can be significantly enhanced with the addition of an adjuvant (Moyle and Toth, 2013) in this matter, the ferritin-based vaccine platform has already shown a favorable immune response against several pathogens (Kanekiyo et al., 2015; Kamp et al., 2020; Swanson et al., 2020) importantly for influenza (Bachmayer et al., 1976; Mett et al., 2008; Szymczakiewicz-Multanowska et al., 2009; Qi et al., 2018; Khalaj-Hedayati et al., 2020), suggesting that the ferritin-based nanoparticle platform could be equally effective in displaying RBD.

The ferritins are a superfamily of well-studied proteins that self-assemble into hollow cage-like structures, which are ubiquitously found in prokaryotes and eukaryotes (Carter Ellenburg et al., 2006; Uchida et al., 2010; Zhang and Orner, 2011). One of the reasons ferritin is so useful for biological applications is its heat and protease resistance properties (Wang Z. et al., 2017; Chakraborti and Chakrabarti, 2019), and the surface, including the internal, external, and subunit interfaces, is susceptible to various modifications (Jin et al., 2019), and also due to its cage-like structure, ferritin is a promising nanoplatform for antigen presentation and immune stimulation (Bhushan et al., 2014; Jutz et al., 2015; Yao et al., 2020; Carmen et al., 2021; Wuertz et al., 2021). Few recent studies explored the utilization of the ferritin and SARS-CoV-2 nanoparticle immunogen in immunized mice and nonhuman primates and demonstrate that the S-domain ferritin nanoparticles elicit broadly neutralizing and cross-reactive antibody responses against SARS-CoV-2, and also SARSCoV-1 ACE2-RBD inhibitory activity was observed (Joyce et al., 2021; Gordon et al., 2022).

Several former studies reported that the ferritin SARS-CoV-2 nanoparticle vaccines are promising in clinical trials (Kanekiyo et al., 2013; Powell et al., 2021; Wang et al., 2021; Wuertz et al., 2021). Also, the correct folding and antigenicity of ferritin surface spikes were confirmed by cryoEM (Powell et al., 2021). However, in the case of SARS-CoV-2, the molecular mechanism of the nanoparticle assemblies and atomistic interactions has not been studied.

The use of glycans in drug design has a long history, and an essential element in developing effective subunit vaccines is the characterization of the glycosylation of viral proteins (Gong et al., 2021). Glycosylation is the most common posttranslational modification that occurs for the virus, and the SARS-CoV-2 proteins, especially the S protein and its receptor ACE2, are densely glycosylated (Shajahan et al., 2020; Woo et al., 2020; Gong et al., 2021; Guo et al., 2021). In several studies, glycosylation has been shown to be essential for protein folding, stability, and ligand binding (Azimzadeh Irani et al., 2017; Azimzadeh Irani, 2018; Azimzadeh Irani and Ejtehadi, 2020; Motamedi et al., 2021; Rahnama et al., 2021). Both N-glycosylation and O-glycosylation occur on the spike protein of SARS-CoV-2, and the latter has a significant effect on virus function and infectivity (Xu et al., 2020; Yasunori et al., 2020; Antonopoulos et al., 2021; Bagdonaite et al., 2021; Reis et al., 2021; Zhang et al., 2021). The results of atomic and molecular dynamics simulations of SARS-COV-2 RBD in complex with ACE2 suggested that O-glycosylation of S494 may result in stronger interactions between RBD-ACE2 and increase viral infectivity (Rahnama et al., 2021).

Considering that the immunogenicity of the nanoparticle vaccine would be the most efficient by adding adjuvants, it can be the best candidate vaccine platform to fight the pandemic. Promoting this vaccine is of great importance in treating and managing COVID-19 and might be practical for similar viral diseases.

In this *in silico* study, two unique facets of vaccine design were used to model improved nanoparticle vaccines. Ferritin nanoparticles were used to stimulate further the immune system attached to the RBD. Each of these ferritin units has an attached RBD that, when aggregated, greatly stimulates the immune system. In this design, the capabilities of glycans were used to bind two proteins that had been previously studied but had not been used for therapeutic purposes.

**FIGURE 1:**
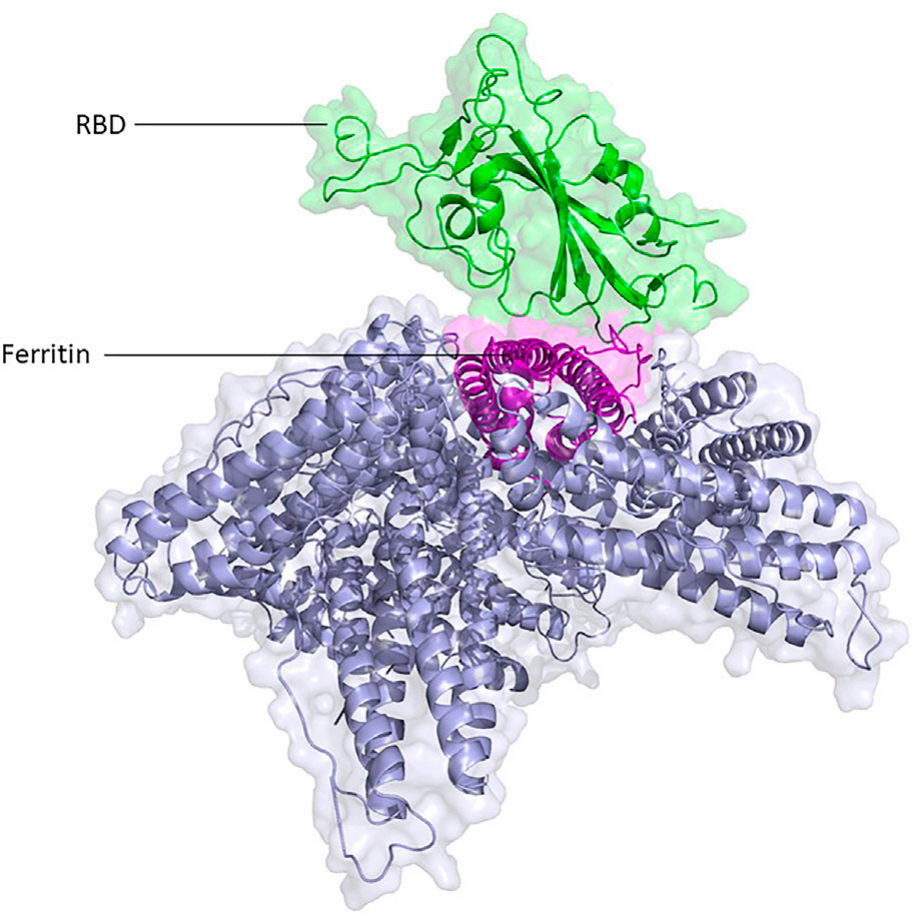
Structural presentation of RBD attached to one unit of ferritin among its eight units. RBD and ferritin are shown in green and purple, respectively. Ferritin self-assembled units are shown in gray.

**FIGURE 2:**
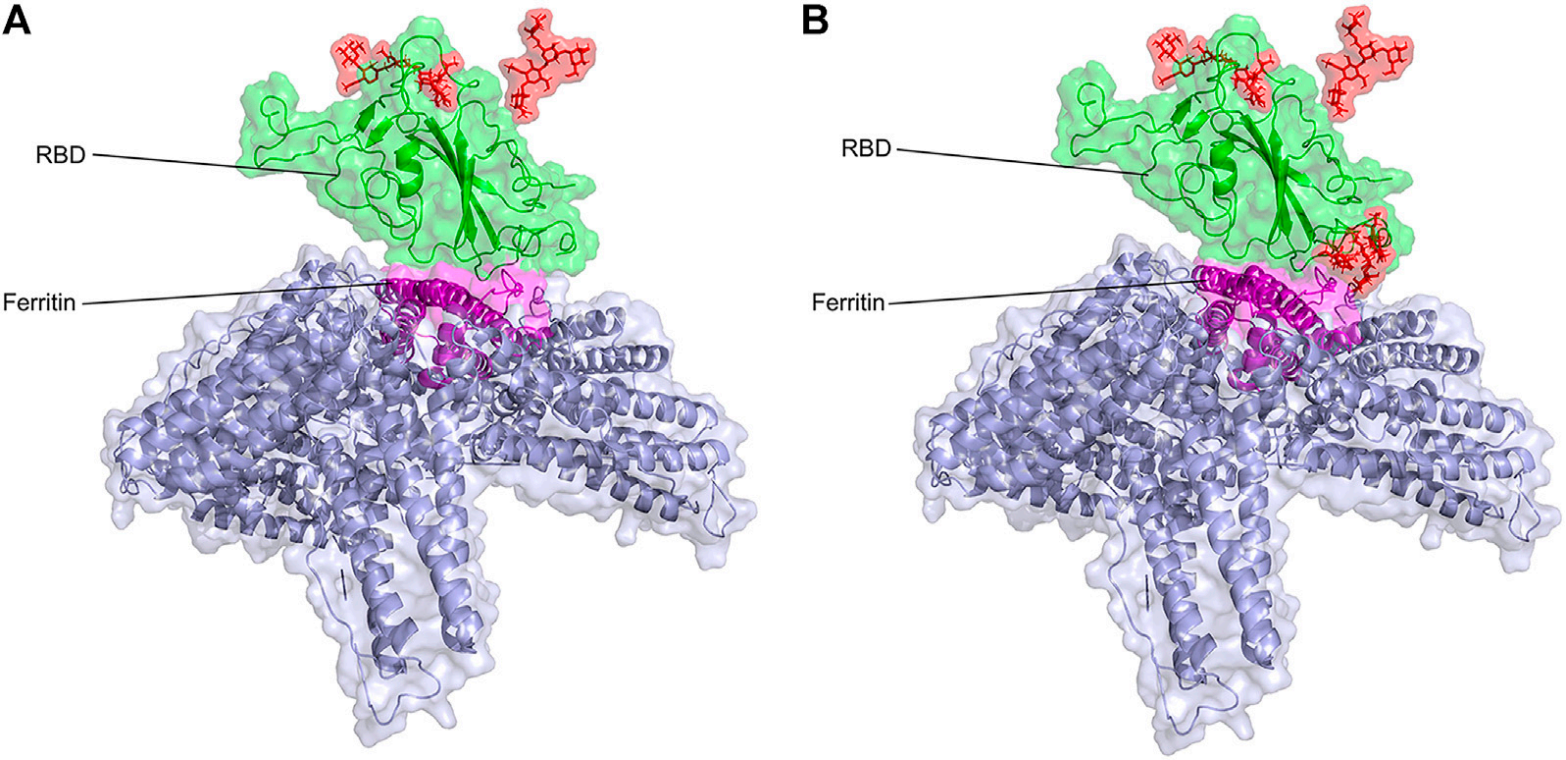
Structural presentation of gRBDfN and gmRBDfN systems in **(A)** and **(B),** respectively. RBD and ferritin are shown in green and purple, respectively. Ferritin self-assembled units are shown in gray. Glycan chains are shown in red sticks.

The atomic model of glycosylated ferritin-RBD nanoparticle vaccine is modeled for the first time, and their interactions are studied at the atomic level (Figure 1). Then, the existing nanoparticle vaccine was upgraded by the addition of O-glycan chains introduced at the RBD–ferritin interface, which increased the binding of the two proteins (Figure 2B).

As this modified nanoparticle vaccine is designed computationally, this construct’s features and general stability are yet to be certified *in vitro* and *in vivo*.

Experimental validation and the use of these findings in vivo can significantly contribute to the ongoing research around viral infections.

## Computational methods

### Construction of the nanoparticle vaccine model

The crystal structure of the SARS-CoV-2 spike receptor-binding domain in complex with ACE2 (PDB ID: 7lo4) (Parker Purcell et al., 2021) was chosen as the model of RBD. This structure, with its high resolution and no missing residues despite the mutation in G485R, which was far from the binding surface of ferritin-RBD, was the best selection of the time. The PDB ID 2fg8 (Wang et al., 2006) crystal structure was used as the model for human L-ferritin.

### Molecular docking of Ferritin-RBD

The selected structures of RBD and ferritin were used for molecular docking with the haddock server (Van Zundert et al., 2016). Several molecular dockings were performed to find the best binding site, conformational state, and binding energy, leaving the ACE2 binding site fully exposed (Table 1). One exposed loop on the ferritin structure (residues:73–91) was selected as the most plausible binding site, which was confirmed by comparing the energetic terms and clustering of the resulting docked poses. The docked pose with the minimum Haddock score and lowest RMSD value (0.3 Å) was considered for further analysis. The poses of complexes generated by Haddock were further processed and visualized with PyMOL to reach a more accurate approximation.

**TABLE 1:** Molecular docking data, represented with the HADDOCK outputs.

NO.	Haddock score	Cluster size	RMSD	vdw energy	Elect energy	Desol energy	Restr energy	Buried surface area	Z-score
**1**	−69.9 +/−1.3	137	15.4 +/−0.2	−38.6 +/−1.1	−118.8 +/−6.3	−9.2 +/−2.3	16.8 +/−14.6	915.7 +/−45.6	−1.8
**2**	−95.0 +/−2.2	63	6.4 +/−0.3	−65.0 +/−3.2	−150.8 +/−19.3	−23.0 +/−2.6	231.2 +/−14.5	1,687.0 +/−39.9	−1.6
**3**	−64.1 +/−4.3	21	11.9 +/−0.1	−89.5 +/−2.0	−174.2 +/−8.8	−9.5 +/−1.8	697.4 +/−69.5	2341.6 +/−89.3	−1.2
**4**	−55.7 +/−3.3	30	14.5 +/−0.1	−79.4 +/−6.1	−243.1 +/−19.2	−9.1 +/−2.3	−9.1 +/−2.3	2388.0 +/−59.5	−1.8
**5**	−94.0 +/−5.9	14	13.8 +/−0.0	−105.2 +/−9.8	−266.0 +/−46.3	−14.6 +/−5.3	789.9 +/−113.2	2841.2 +/−129.5	−2.5
**6**	−83.4 +/−6.0	33	23.4 +/−0.0	−28.9 +/−6.3	−313.0 +/−39.9	7.0 +/−3.4	11.7 +/−4.0	1,301.9 +/−83.9	−0.9
**7**	−83.9 +/−5.6	16	12.1 +/−0.1	−55.1 +/−6.6	−341.8 +/−48.9	13.1 +/−2.5	264.6 +/−32.7	1809.4 +/−41.0	−1.4
**8**	−91.0 +/−3.3	15	5.7 +/−0.3	−57.6 +/−4.8	−57.6 +/−4.8	−57.6 +/−4.8	146.4 +/−68.1	1,660.9 +/−81.8	−2.4
**9**	−75.6 +/−12.2	8	7.6 +/−0.2	−56.8 +/−3.1	−207.9 +/−36.6	0.5 +/−3.3	222.6 +/−13.1	1,533.6 +/−82.2	−2.2
**10**	−97.8 +/−4.1	13	1.8 +/−1.4	−48.5 +/−10.5	−364.9 +/−45.7	−2.6 +/−2.9	262.7 +/−57.6	1765.5 +/−177.2	−1.7
**11**	−114.2 +/−7.3	35	0.8 +/−0.5	−60.5 +/−8.2	−371.4 +/−25.7	−2.7 +/−1.3	233.0 +/−44.3	2024.0 +/−107	−2.1
**12**	−80.5 +/−5.1	37	0.3 +/−0.2	−51.7 +/−4.0	−300.1 +/−26.3	1.3 +/−3.2	299.6 +/−15.1	1,576.7 +/−86	−1.8
**13**	−89.9 +/−21.1	9	0.5 +/−0.3	−76.2 +/−16.7	−289.4 +/−24.9	2.9 +/−2.7	413.1 +/−16.2	2128.9 +/−56	−1.8
**14**	−86.7 +/−4.1	81	9.2 +/−0.1	−54.0 +/−6.4	−284.4 +/−11.2	−2.5 +/−1.4	267.0 +/−58.9	267.0 +/−58	−1.9
**15**	−64.8 +/−5.1	33	6.6 +/−0.5	−45.2 +/−6.0	−220.9 +/−10.4	1.6 +/−0.6	230.1 +/−21.1	1,307.8 +/−63	−1.1
**16**	−80.1 +/−6.4	18	11.2 +/−0.2	−55.3 +/−8.7	−262.2 +/−14.9	−1.7 +/−2.4	293.6 +/−63	293.6 +/−63	−1.4

### Model construction of the modified nanoparticle

The RBD sequence selection is based on the experimentally constructed nanocages (Kalathiya et al., 2021; Powell et al., 2021). They used sequence optimization for creating the nanoparticle. The loop was modified using the Python scripts in MODELLER (version 10.0) (Martí-Renom et al., 2000; Eswar et al., 2006), and 100 models were generated; the one with the lowest Z-DOPE scores was retained. Loop structure with Ser-Ser-Thr-Ser-Ser amino acids was inserted into the RBD crystal structure using the builder module of PyMOL. This was followed by 1,000 steps of Steepest Descent/Conjugate Gradient minimization to relax the structure by UCSF chimera.

The modified RBD model was glycosylated with GLYCAM builder (GLYCAM, 2020) at the Asn343 native N-glycosylation site with (Man1-3 [Man1-6]Man1-4GlcNAc1-4GlcNAc1-OH), and the Ser494 native O-glycosylation site with (Gal1-4GlcNac1-4Gal1-4GlcNAc1-6 [Gal1-3]GalNAc1-OH) and core (GalNAc-Gal) (Gong et al., 2021) was used for glycosylation of three sites in the loop structure, and also the GLYCAM server minimized the models.

### Molecular dynamics simulations and analyses

Three systems were set up, including the unglycosylated RBD-ferritin (Figure 1), glycosylated RDB-ferritin with the two native chains of N- at Asn343 and O-glycans at Ser494 of RBD, and the modified glycosylated vaccine model with the native glycans and three extra glycan chains at Ser386, Thr387, and Ser388 of RBD (Figure 2).

All molecular dynamics simulations were carried out with the AMBER16 package (Case et al., 2016). The parameters and topology files were generated using the Xleap module of AMBER16 (Pearlman et al., 1995). Protein residues were presented with an Amber ff14SB force field (Maier et al., 2015). The systems constructed above were immersed in a box of TIP3P waters with at least an 8 (Å) boundary around any atom, and the systems were neutralized with counterions.

Heating up of the systems was performed up to 300 K (0–100 K, 100 K–200 K, and 200–300 K) for 100 ps, followed by equilibration of the whole complex for 250 ps Three replicates of ns production runs were performed on each system utilizing the isothermal-isobaric ensemble (NPT) scheme. A weak-coupling algorithm (Berendsen et al., 1984) was utilized to constant pressure dynamics *via* the reference pressure set to 1 bar and maintained with 1-ps relaxation time. Langevin dynamics (Pastor et al., 1988; Loncharich et al., 1992) with a collision frequency of 1 ps−1 K was used to maintain the temperature at 300 K. The nonbonded interactions (electrostatics and VDW) were computed with a 9 (Å) cutoff value, and the Particle Mesh Ewald (PME) method was carried out to simulate the long-range electrostatic interactions. The SHAKE algorithm was applied to constrain the bonds involving hydrogens, enabling a time-step of 2 fs to be used in the simulations.

RMSD, RMSF, PCA, and cross-correlations of the CA atoms were calculated in the Bio3D package (Grant et al., 2006) in R for each model over the 100 ns of simulation time. All the trajectories were fitted to the starting conformation.

Visual molecular dynamics (VMD) (Humphrey et al., 1996) was used to visualize the correlated and anti-correlated motions. Secondary structure timeline analyses were calculated for 200 frames over VMD’s 100 ns of simulation time.

### Indicator immune response simulation

The C-IMMSIM server (Rapin et al., 2010; Castiglione et al., 2021) was used for performing the immune simulation of the modified RBD-ferritin vaccine in order to characterize the immune response profile and immunogenicity of the antigenic peptides. The experiment was performed by inserting FASTA sequences of vaccine constructs, and the entire simulation ran for 1,100-time steps which are about 12 months (a time step is about 8 h). Random seed and simulation volume were set as 12,345 and 50, respectively. Three *in silico* injections were given at the time steps of 1, 84, and 168 with no LPS and maintained a minimum of 30 days of the time interval between two injections.

## Results

### Dynamics of the refined RBD-ferritin nanoparticle

Henceforth, the RBD-ferritin nanoparticle, glycosylated RBD-ferritin nanoparticle, and glycosylated modified RBD-ferritin nanoparticle systems will be referred to as RBDfN, gRBDfN, and gmRBDfN, respectively.

**FIGURE 3:**
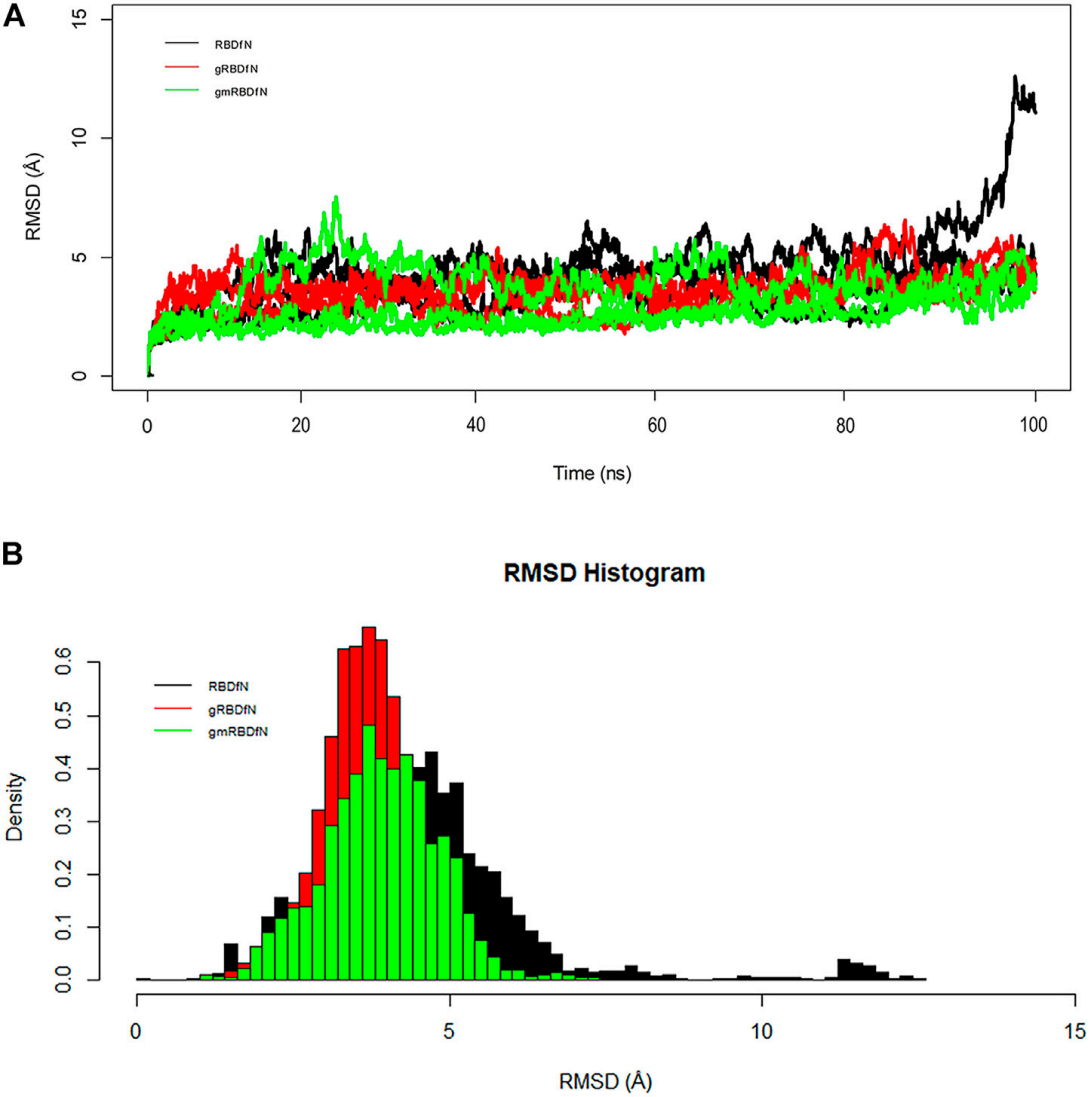
**(A)** RMSD of all systems is calculated over 100 ns MD simulations. The structural distance of CA atoms calculated for all replicates of RBDfN, gRBDfN, and gmRBDfN systems are shown in black, red, and green, respectively. **(B)** RMSD distribution of CA atoms is calculated over 100 ns MD simulations for the first replicates. RBDfN, gRBDfN, and gmRBDfN systems are shown in black, red, and green, respectively.

The overall RMSD plot shows that the gmRBDfN model is the most stable of the three systems, while the gRBDfN model shows a comparable RMSD range to the gmRBDfN model, and the RBDfN model is the least stable system (Figure 3A).

Average RMSD values for the RBDfN, gRBDfN, and gmRBDfN are 4 (Å), 3.5 (Å), and 3 (Å), respectively. The average RMSD values also show that the gmRBDfN system is the most stable. This observation was expected as several other experimental, computational, and structural studies have reported that glycosylation contributes to increasing the stability of protein structures (Solá and Griebenow, 2009; Azimzadeh Irani, 2018; Azimzadeh Irani and Ejtehadi, 2019; Motamedi et al., 2021).

The histogram of the overall RMSD shows that the lowest RMSD belongs to the gmRBDfN system, while the RMSD of the gRBDfN system is more persistent. Among these models, the RBDfN model has the most dispersed RMSD values. Furthermore, the gRBDfN and gmRBDfN plots are quite comparable. While their RMSD peaks are 0.5 (Å) different, the gRBDfN model has a smaller RMSD value (Figure 3B).

**FIGURE 4:**
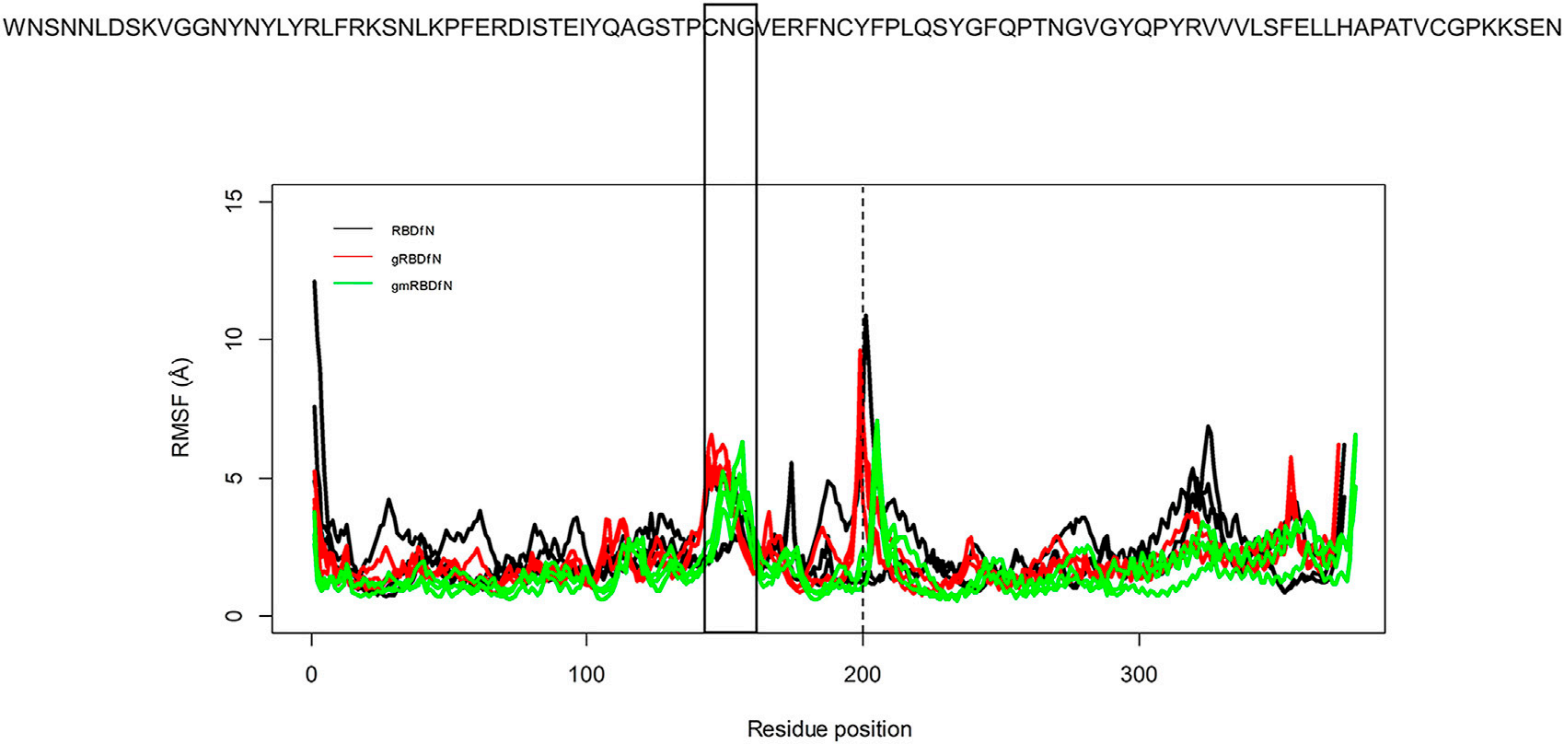
RMSF of all systems is calculated over 100 ns MD simulations. Fluctuations of CA atoms calculated for all replicates of RBDfN, gRBDfN, and gmRBDfN systems are shown in black, red, and green, respectively. The dashed line shows the boundary between the RBD and ferritin amino acids. In the upper panel, reference amino acid sequences are the amino acids of the RBD region.

The RMSF was calculated to explore the fluctuations of the CA atoms in all systems (Figure 4). Average RMSF values for RBDfN, gRBDfN, and gmRBDfN are 2 (Å), 1.8 (Å), and 1.5 (Å), respectively. Similar to what was observed in the RMSD plot, the gmRBDfN system shows the lowest fluctuations, followed by the gRBDfN and then RBDfN systems.

**FIGURE 5:**
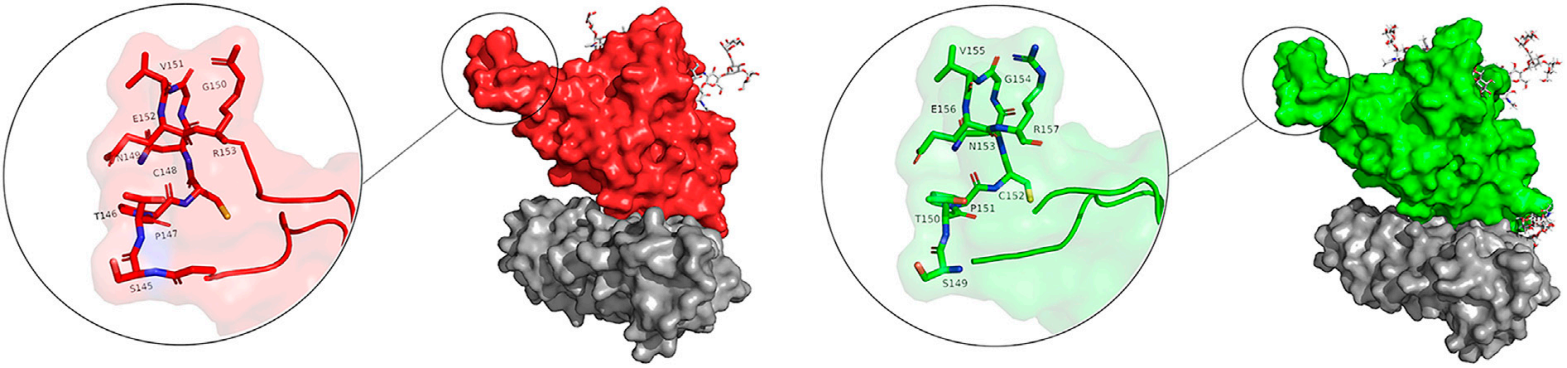
Flexible loop within the RBD structure is shown in the sticks. Picture **(A)** shows the gRBDfN, and picture **(B)** shows the gmRBDfN system. Ferritin is shown with a gray surface, and the RBD of gRBDfN and gmRBDfN are shown with red and green surfaces, respectively.

There are two clear peaks in the RMSF plots of the glycosylated systems (gRBDfN and gmRBDfN). One in residues 480–482 of RBD includes cysteine, asparagine, and glycine amino acids, corresponding to a flexible loop region on the protein surface (Figure 5A). Visualization of the dynamics shows that this loop is distant from the binding interface and glycosylation sites. Similar fluctuations are seen in the C-terminus of the RBD structure, which is expected to be more flexible (Figure 4).

**FIGURE 6:**
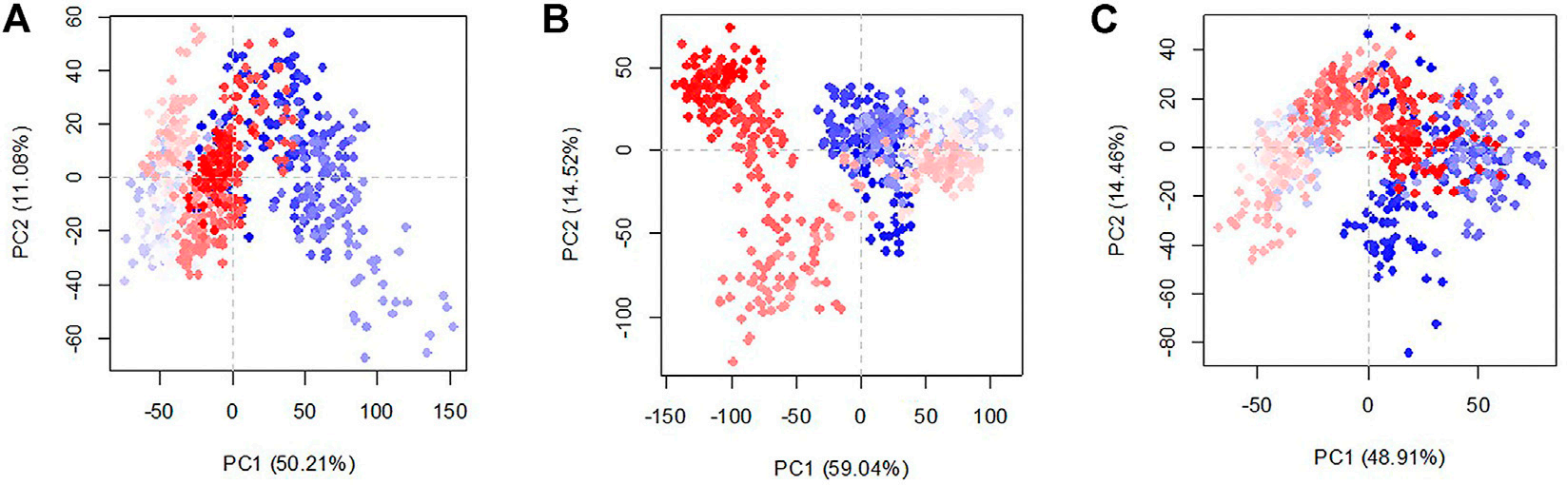
Characteristic conformation variance of all systems calculated over 100 ns MD simulations. Shades of red and blue spots present conformational changes. RBDfN, gRBDfN, and gmRBDfN systems are shown in pictures **(A)**, **(B)**, and **(C)**, respectively.

To clarify the effect of glycans in the conformation of the structures in the three existing models, the principal component analysis was studied (Figure 6). The percentage of variance captured by PC1 and PC2 of the systems are RBDfN 50.2 and 11.1%, gRBDfN 59 and 14.5%, and gmRBDfN 48.91 and 14.46%, respectively. The eigenvalue rank plot shows that the first two principal components of RBDfN, gRBDfN, and gmRBDfN cover 61, 73, and 63% of the total variance, respectively. The reduction in the variance of the gmRBDfN system compared to the RBDfN and gRBDfN shows that the fluctuations of the gmRBDfN system are dampened upon glycosylation. Decreased conformation variance suggested stabilizing the role of glycans at modified sites in the gmRBDfN system.

### Stabilization of the RBD within the refined nanoparticle

**FIGURE 7:**
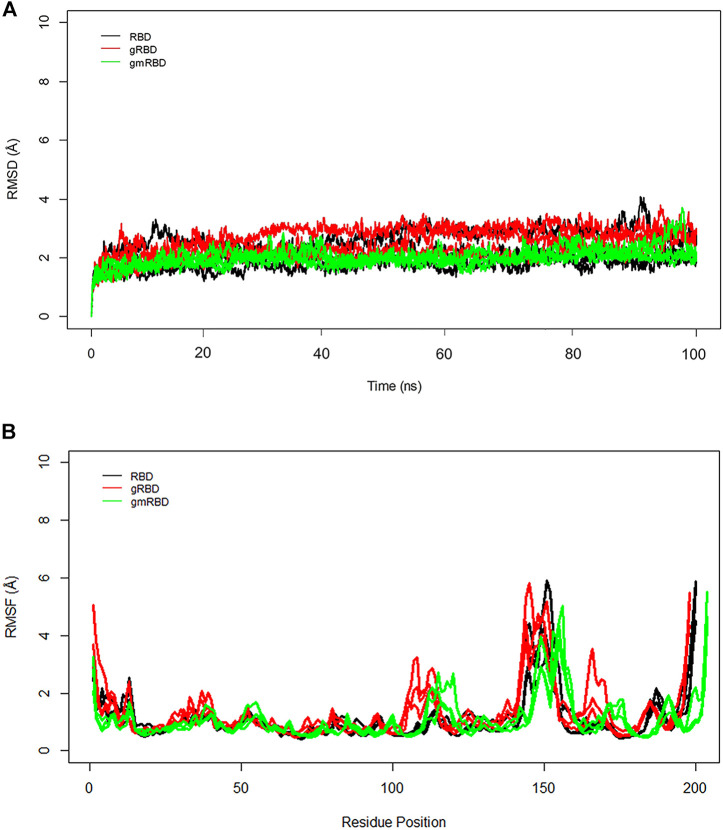
**(A)** RMSD of all systems is calculated for RBD over 100 ns MD simulations. The structural distance of CA atoms calculated for all replicates of RBD, gRBD, and gmRBD systems are shown in black, red, and green, respectively. **(B)** RMSF of all systems is calculated for RBD over 100 ns MD simulations. Fluctuations of CA atoms calculated for all replicates of RBD, gRBD, and gmRBD systems are shown in black, red, and green, respectively.

To better understand the RBD interactions with ferritin, RMSD and RMSF plots were calculated separately for the RBD and ferritin in the complex. The general trend is in good agreement with the overall RMSD plots. The gmRBDfN is the most stabilized system with the smallest RMSD values along with the RBD (Figure 7A), while the gRBD is the most flexible of all systems because of the motions of the loops in its structure which causes multiple peaks in the RMSF plot (Figure 7B). The average RMSD values for RBD, gRBD, and gmRBD are 2.1 (Å), 2.5 (Å), and 1.9 (Å), respectively.

In the RMSF plot of RBD (Figure 7B), high peaks are observed close to residues 480–482, indicating a loop’s position. This flexible loop has the highest RMSF value in all systems. However, it shows the lowest flexibility in the gmٍRBDf model due to several RBD–ferritin interactions that result in more excellent overall structural stability. This loop is far from the ferritin interface and out of glycans’ reach, yet it is more stable due to the overall increased stability of the gmRBDfN’s complex (Figure 5B, Figure 7B).

**FIGURE 8:**
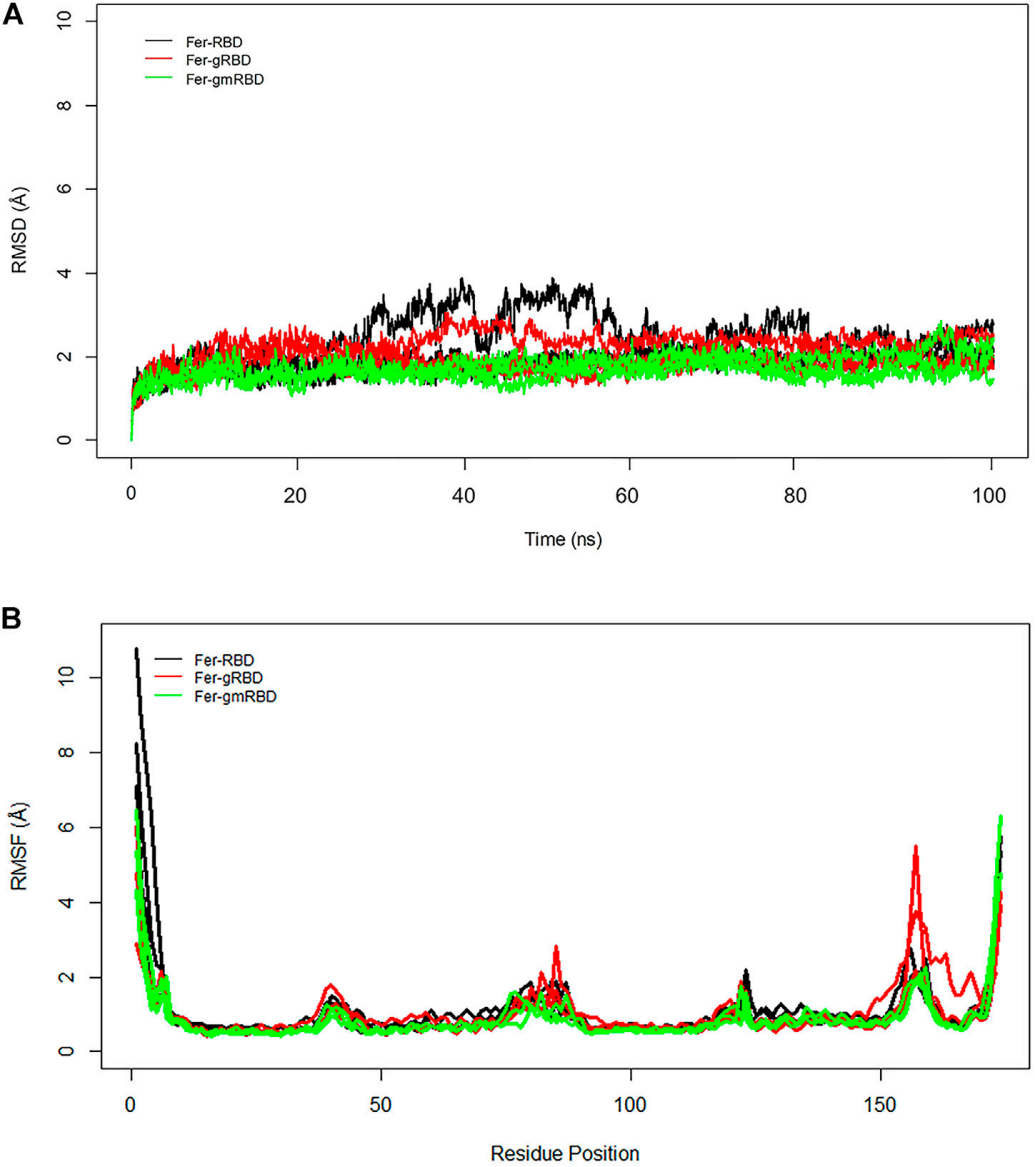
**(A)** RMSD of all systems is calculated for ferritin over 100 ns MD simulations. The structural distance of CA atoms calculated for all replicates of Fer-RBDfN, Fer-gRBDfN, and Fer-gmRBDfN systems are shown in black, red and green, respectively. **(B)** RMSF of all systems is calculated for ferritin over 100 ns MD simulations. Fluctuations of CA atoms calculated for all replicates of Fer-RBDfN, Fer-gRBDfN, and Fer-gmRBDfN systems are shown in black, red, and green, respectively.

RMSD plots of ferritin show that the ferritin structure is more stable in the gRBDfN and gmRBDfN systems as opposed to the RBDfN system (Figure 8A). The average RMSD values for the Fer-RBDfN, Fer-gRBDfN, and Fer-gmRBDfN are 2.02 (Å), 2.01 (Å), and 1.7 (Å), respectively. Thus, glycosylation seems even to increase the stability of the ferritin cage in the gmRBDfN nanoparticle. Glycosylation in the gRBDfN system is not as dense as gmRBDfN. Yet, the stability of both glycosylated systems increases compared to that of the RBDfN with no attached glycans.

The RMSF plots of ferritin show two noticeable peaks at residues 73–91, the RBD–ferritin interface, and at 153–160 residue position, which is located in the C-terminal of ferritin (Figure 8B).

**FIGURE 9:**
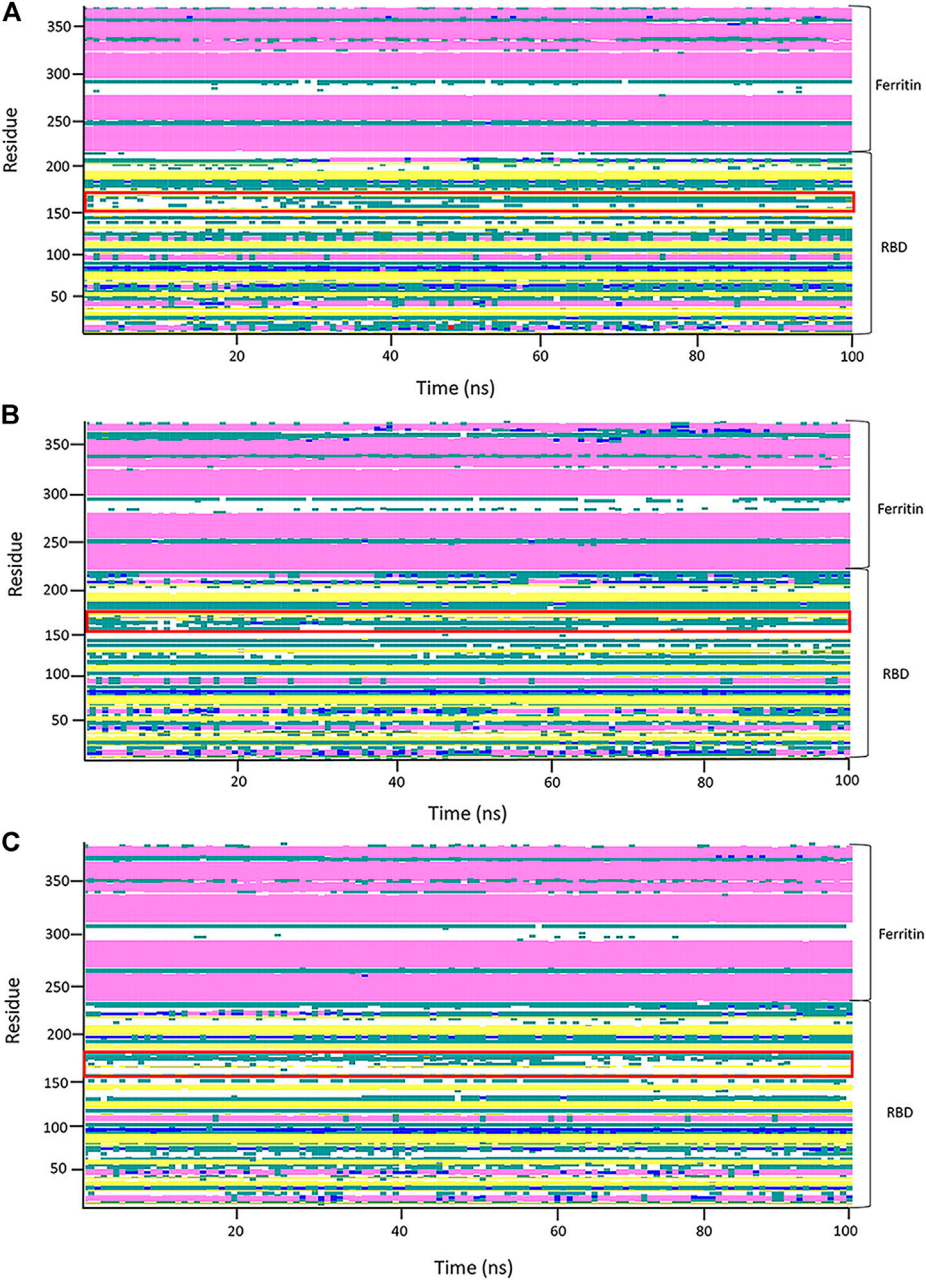
Analysis of the secondary structure through 100 ns of molecular dynamics (MD) simulation of the first replicates of systems. Secondary structure analyses of the RBDfN, gRBDfN, and gmRBDfN systems are shown in **(A)**, **(B)**, and **(C)**, respectively. A flexible loop of structures is shown within the red box.

A secondary structure timeline was calculated for the RBD–ferritin protein structure (Figure 9). The secondary structure of RBD has a lot of structural changes due to its many loops that reduce the structural stability. Contrary to what is seen in the RBD secondary structure timeline, the ferritin’s secondary structure shows no significant structural changes in the ferritin structure.

In general, the stability of the ferritin in all three systems is very high, and in none of them do its dynamics undergo profound changes. All of these investigations are supported by the RBD and ferritin RMSF plots (Figure 7B, ). By tracing the flexible loop in the secondary structure timeline, it could be observed that its stability increases by increasing the number of the attached glycans to the nanoparticle. With gmRBDfN, gRBDfN and RBDfN show the highest stability. These observations give us another piece of evidence of the role of glycans in increasing the stability of the ferritin nanoparticle vaccines.

### Detailed atomistic interactions of the refined RBD-ferritin in the gmRBDfN and gRBDfN systems

**FIGURE 10:**
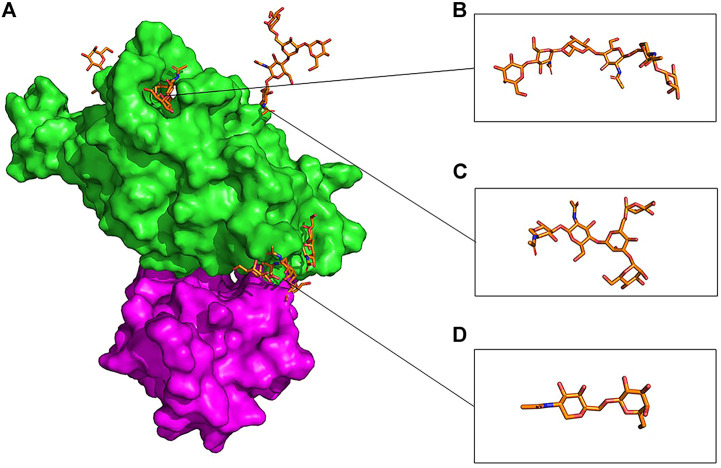
Overlaid presentation of glycosylated RBD in the gmRBDfN system is shown on the green surface, and ferritin is shown on the purple surface in picture **(A)**. S494 O-glycan is shown in picture **(B)**, N343 N-glycan is shown in picture **(C)**, and O-glycan core is shown in picture **(D)** with orange sticks.

**FIGURE 11:**
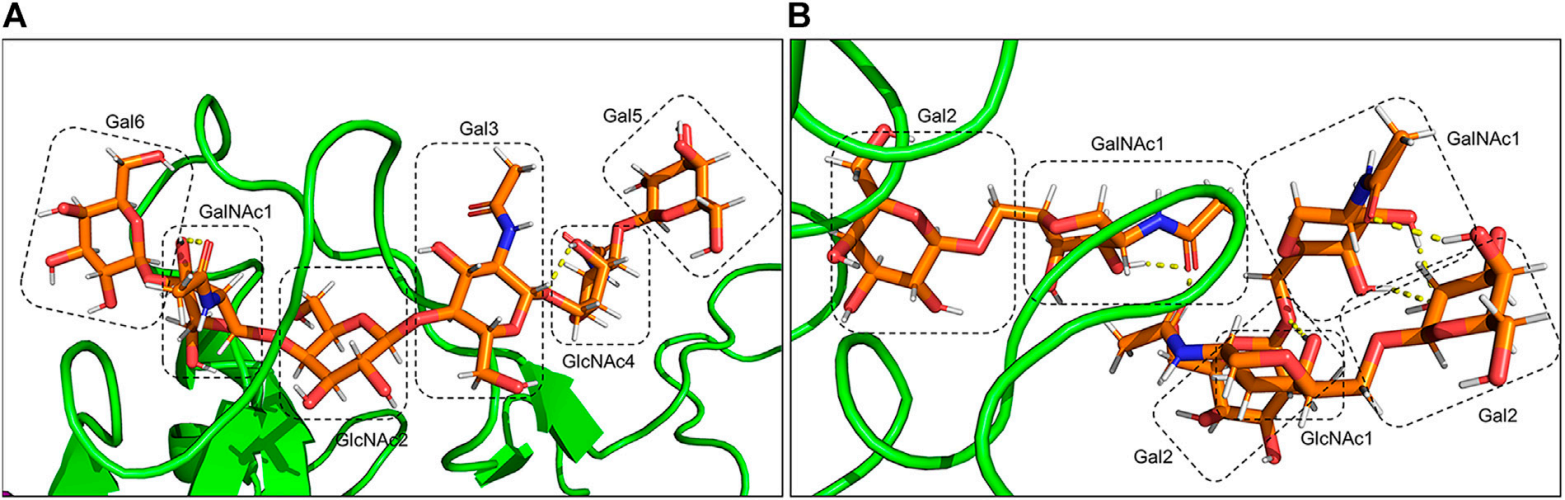
Hydrogen bonds between O-glycans of the gmRBD are shown in yellow dashed lines. **(A)** O-glycan attached to Ser494, and **(B)** additional O-glycans attached to Ser386,388 and Thr387.

The presence of the O-glycan at S494 of the RBD interface with ACE2 (Figure 10) increases movement and flexibility, which was reported in other studies (Rahnama et al., 2020). At the same time, the presence of additional glycans in the complex of the gmRBD causes ferritin and RBD to stick together like glue (Figure 11). Two previous studies have reported this glue-like function of the glycans in protein–protein interactions (Azimzadeh Irani and Ejtehadi, 2020; Motamedi et al., 2021). These observations are consistent with the increased stability of the refined nanoparticle shown in the RMSD, PCA, and secondary structure plots (Figure 3A, Figure 6, Figure 9).

At the atomic level, the gRBDfN model has an N-glycan attached to the Asn343 and O-glycan attached to the Ser494 (Figure 11). The gmRBD model has the previous glycans in the gRBDfN model and three additional O-glycans in the RBD–ferritin interface (Figure 10). All glycans establish interactions in gRBDfN and gmRBDfN structures, which are hydrogen bonds. The glycans attached to these amino acids were characterized by several intra-glycan hydrogen bonds and several hydrogen bonds with RBD structures. O-glycan had inter-and intra-hydrogen bonds in itself, but N-glycan had no intra-hydrogen or inter-hydrogen bonds.

**FIGURE 12:**
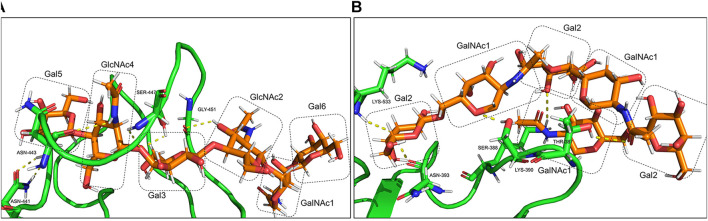
Intra-hydrogen bonds between glycans and the amino acids of gmRBD are shown in yellow dots. **(A)** O-glycan attached to Ser494 and **(B)** O-glycans core attached to Ser386,388 and Thr 387.

O-glycan inter-hydrogen bonds were formed between GalNAc1-Gal6 and GlcNAc4-Gal3 (Figure 11A). Intra-hydrogen bonds between O-glycan and the RBD structure were formed between Gal6-Asn441, GalNAc1-Asn443, Gal3-Ser447, and GlcNAc2-Gly451 (Figure 12A). In the gmRBDfN model, O-glycan cores had hydrogen bonds between glycans and intra-hydrogen bonds between Gal2-Lys533 and Asn393, GalNAc2-Ser388, Gal2-Lys390, and GalNAc1-Thr387 (Figure 11B, Figure 12B). Intramolecular hydrogen bonds in the glycans attached to Ser494, Ser386, Ser388, and Thr387 appear to impart stability to the tertiary structure of the complex. All these hydrogen bonds result in stability of the structure that has been shown in previous investigations. This could contribute to the most reduced flexibility of the gmRBDfN system. O-glycans are stabilized by interactions with each other mediated by various intramolecular hydrogen bonds; however, they are not persistent.

### Strengthened RBD–ferritin interaction in the refined nanoparticle

**FIGURE 13:**
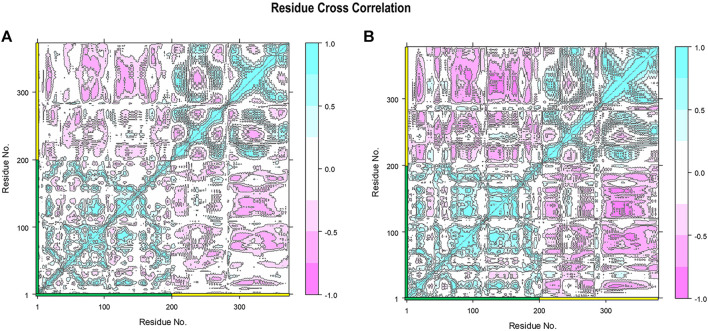
Residue cross-correlation matrix of gRBDfN and gmRBDfN is calculated over 100 ns MD simulations for CA atoms. Shades of red and blue spots present the atom’s correlated and anti-correlated motions, respectively. The gRBDfN and gmRBDfN systems are shown in **(A)** and **(B)**. The amino acid residues of RBD and ferritin are marked on the *x* and *y* axes with green and yellow, respectively. The values of the correlations range from -1 to 1 as shown in the bar on the right.

To specifically show the effect of stabilized RBD–ferritin interactions in the gmRBDfN system, the cross-correlation between the fluctuations of the CA atoms was mapped for the dynamics of all systems (Figure 13 and Supplementary Figure S1). The positive and negative values represent the correlated and anti-correlated motions, respectively. Within the RBD and ferritin structures, the correlated motions are increased in the gmRBDfN system compared to the gRBDfN and RBDfN (Figure 13 and Supplementary Figure S1), which shows the increased stability of the RBD and ferritin in the modified system. This is consistent with the findings from the RMSF analyses, which showed decreased fluctuations of both subunits and the overall vaccine construct (Figures 4, 7, 8).

**FIGURE 14:**
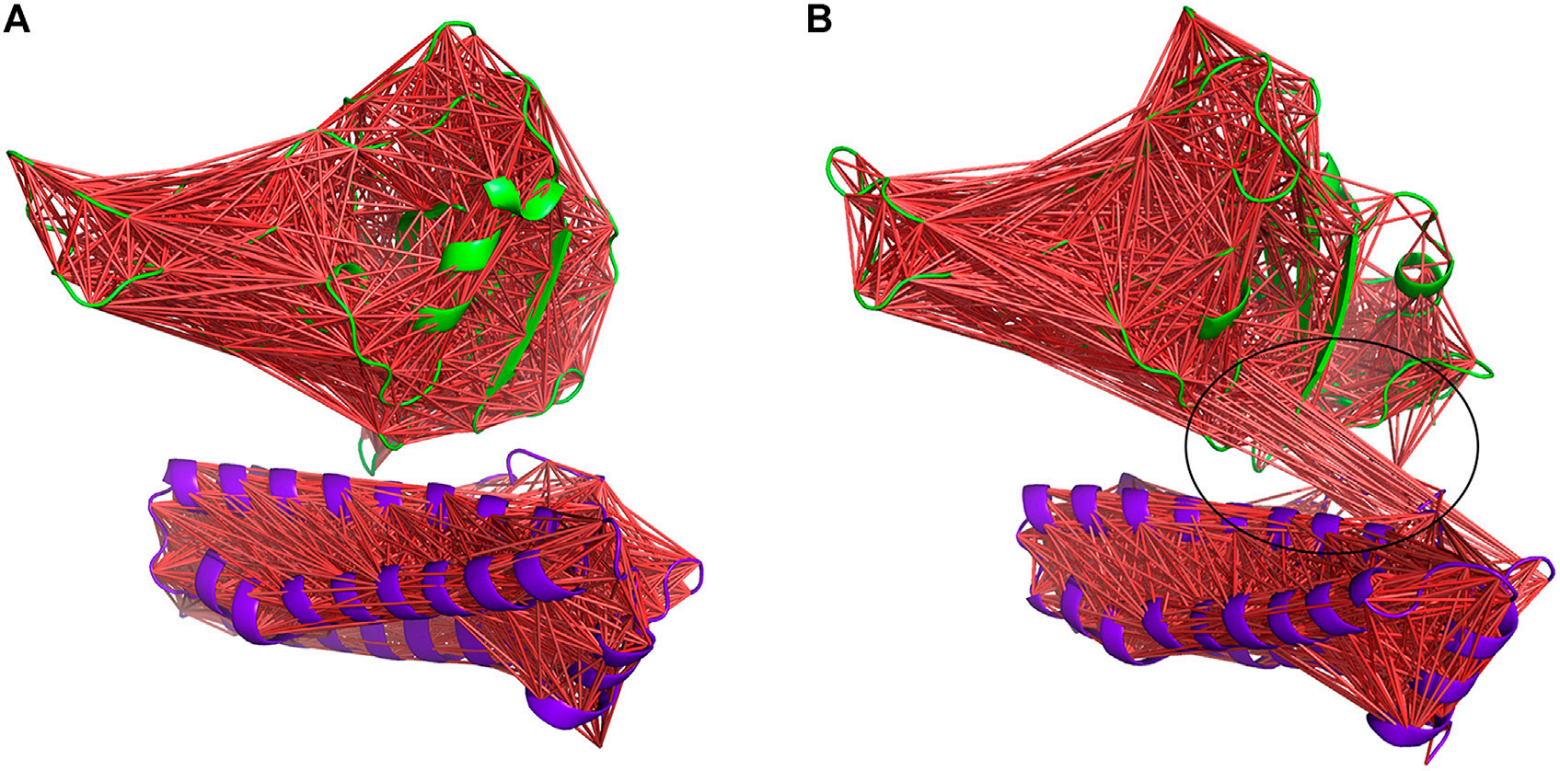
DCCM of gRBDfN and gmRBDfN is calculated over 100 ns MD simulations. gRBDfN and gmRBDfN are shown in **(A)** and **(B)**, respectively. Dark red lines show anti-correlated motions between RBD in green and ferritin in purple cartoons. Specific anti-correlated motions between the ferritin and RBD are shown in the circle in picture **(B)**.

However, the most interesting alteration has occurred between the RBD and ferritin in the gmRBDfN system (Figure 13). Anti-correlated motions in residues 30 to 50 of ferritin structure and residues 200 to 330 of RBD structure are noticeably more pronounced in the gmRBDfN system. Anti-correlated fluctuations between the RBD and ferritin atoms show that ferritin and RBD were moved toward each other and got closer in the gmRBDfN than in the gRBDfN and RBDfN systems (Figures 13, Figure 14 and Supplementary Figure S1), which leads to a stronger interaction between RBD and ferritin in the refined nanoparticle.

### Immune simulation of the refined RBD-ferritin model vaccine

**FIGURE 15:**
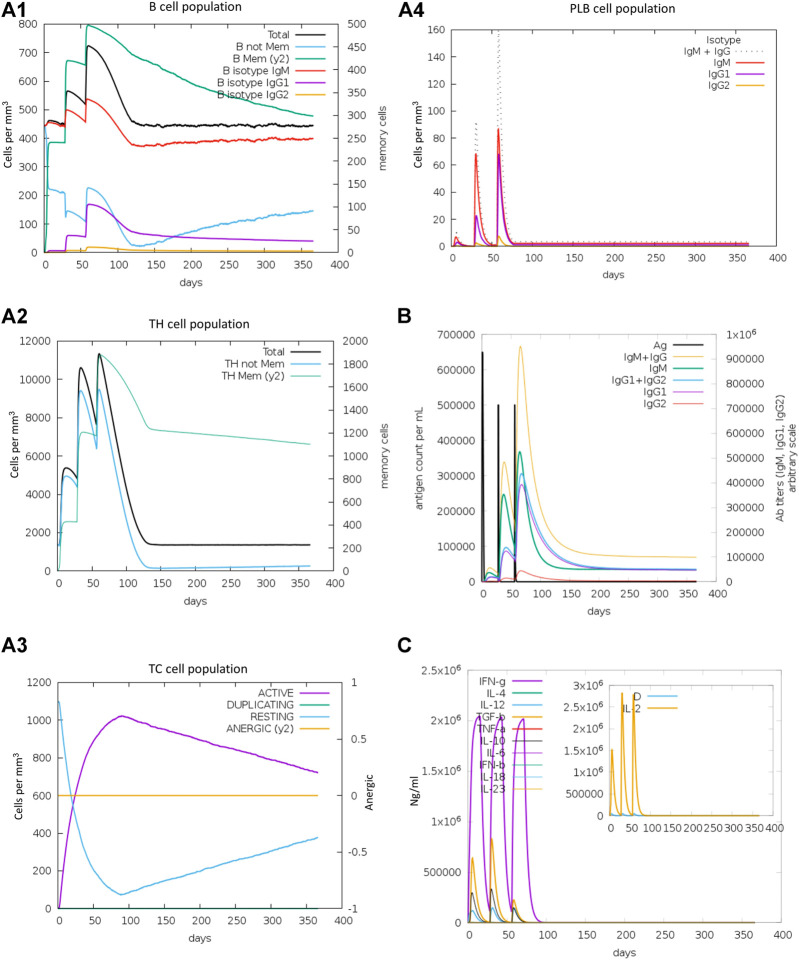
Immune response simulations of refined RBD–ferritin nanoparticle vaccine. **(A1)** B cell population. **(A2)** TH cell population. **(A3)** CTL population. **(A4)** Plasma B cells. **(B)** Antigen and immunoglobulins. **(C)** Cytokines and interleukins.

The immunogenic profile of the refined RBD-ferritin vaccine candidate was obtained from the C-IMMSIM server. The vaccine is injected in three doses over a period of 2 months and 30 days of time intervals between two injections. It found that our vaccine candidates could elicit both humoral and cellular-mediated immune responses (Figure 15). In plots A1 and A2, the B cell population and the total number of lymphocytes along with the CD4^+^ T cell are shown, respectively, which shows the increase in cell population after each vaccine dose. Panel A3 shows the CD8^+^ T cell population per cell state, showing the active cell population is higher than the anergic cells, and panel A4 shows the plasma cell population, which reaches a higher cell concentration (85 cells per mm) after the third dose. Panel B shows that the secondary and tertiary immune responses eliminate the antigen on a shorter timescale due to the presence of memory cells ready to react. An increasing trend of IgM and IgG antibody titer was observed after the third injection, while the antigen level was decreased. It was also shown that levels of cytokines such as IFN-γ and IL-2, which are essential for inhibition of viral replication and T-cell-mediated immunity, are elevated. Antigenic molecules were found to be cleared off after three doses of vaccination; at the same time, the B and T memory cell population increased to the maximum of 800 cells/mm3 and 11,000 cells/mm3, respectively. This makes the construct RBD-ferritin a suitable nanoparticle vaccine candidate.

## Discussion

COVID-19 is a severe and dangerous infectious disease with symptoms similar to SARS, including fever, cough, and fatigue. It is a significant threat to the health and safety of the world and must be prevented from spreading. Ever since the pandemic of COVID-19 began, many efforts have been made to find a cure for this deadly virus. Vaccines have been more effective among all approaches against coronavirus, especially nanoparticle vaccines that produce long-lasting immune responses. The RBD of infusion spike protein contains multiple conformation-dependent epitopes and is the main domain that induces neutralizing antibody and T-cell immune responses against SARS-CoV-2 infection (Qin et al., 2005; He et al., 2006), making it an essential target for vaccine development. In addition, immune responses of cellular and humoral immunity were simulated in three injections over 2 months. Cellular immune cells were increased after each injection, and humoral immunity, including IgG and IgM, shows that there are enough antibodies that can eliminate the antigen after three doses. Reliability of the proposed RBD–ferritin interface was also investigated by examining the mutations that occurred in the spike protein of variants of concern, including Alpha, Beta, Gamma, Delta, and Omicron. It was noticed that none of the mutations exist in the proposed ferritin-binding interfaces. Although most of the mutations, especially the omicron variant mutations, have occurred in the RBD region, and some are near the proposed binding interface, such as G339D and S371L (Alkhatib et al., 2022), they do not disrupt the selected binding surface. Also, according to the predictions that have been made about the mutation sites in SARS-CoV-2 (Chen J. et al., 2020) conducted on the binding affinity of RBD and ACE2 and the alignment comparison of the spike protein sequence in five closely related species including SARS-CoV, bat coronavirus RaTG13, bat coronavirus BM48-31, and bat coronavirus CoVZC45, it can be predicted that the next mutations will probably occur at Y489 and T500 sites due to the non-conservative nature of these residues. But, these plausible mutations are located on the Receptor Binding Motif (RBM) and are far from the ferritin-binding interface. Therefore, the proposed RBD–ferritin interface would not be negatively affected.

Ferritin nanoparticles are of significant interest for developing new vaccines because they self-assemble into stable structures that display RBD protein on their surface (Cho et al., 2009). Coronavirus spike glycoprotein is highly glycosylated by the host cell, generally containing N-linked and O-linked glycosylation sites on each spike trimer. O-glycans, specifically which are involved in protein stability and function on the spike protein, are suggested to play roles in the infectivity of the virus (Bagdonaite and Wandall, 2018; Andersen et al., 2020). As glycosylation can influence the proper folding and stability of viral antigens, it is noteworthy that expression was achieved in mammalian cells, allowing spike proteins to be produced with native-like glycosylation (Watanabe et al., 2020). However, glycans have been proposed for drug and antibody design, but there has been no successful design of this kind before. The ultimate objective of the in silico analyses performed here is to enhance the efficiency of the currently applied ferritin nanoparticle vaccines and pave the path to further experimental assays. Even though ferritin nanoparticle vaccines have already shown promising results in the clinic, currently, there is no biological validation on the construct preparation and purification of the modified nanoparticles proposed here. Such properties and overall stability of these constructs are yet to be approved in vitro and in animal models.

This work is based on existing glycosylation and nanoparticle vaccine experimental and computational works (Liu et al., 2014; Sliepen et al., 2015; Wang L. et al., 2017; Rahnama et al., 2020; Kalathiya et al., 2021; Powell et al., 2021). Herein, a more stable nanoparticle vaccine was designed by modifying the specific loop of RBD near the ferritin interface with a glycan coat. MD simulations of the nonglycosylated, fully glycosylated, and modified glycosylated RBD bound to the ferritin unit revealed a detailed picture of the role of glycans in constructing nanoparticle vaccines. According to the analysis of trajectories, it is clear that more glycosylation increases the stability of the gmRBDfN than that of the gRBDfN system. Previous findings have proposed that glycosylation can be useful in vaccine design (Chang and Zaia, 2019; Hariharan and Kane, 2020; Liao et al., 2021; Schön et al., 2021; Ozdilek and Avci, 2022), yet to the best of the author’s knowledge, there has been no glyconanoparticle vaccine design before this work. The simulations showed that this modification could not only be applied to the structure of RBD on the ferritin cage without affecting the stability but also it enhances the stability of the entire system. This proposed modified nanoparticle could boost the immune response due to its increased stability compared to the currently designed nanocages. The modified glycosylation sites are distant from ACE2 binding epitopes of RBD (Figure 2). Also, as the modifications occur at the self-assembled ferritin binding interface prior to nanocage release, the immune response is unlikely to be altered. These findings proved that ferritin glycovaccine is a promising scaffold for all microbial pathogens with a similar arrangement.

## Conclusion

RBD-ferritin nanoparticles are one of the most effective vaccines that provide optimal immunity to the virus in a single dose. In this work, molecular modeling and MD simulations combined the self-assemble ferritin nanoparticle scaffold with glycosylation to improve the currently available ferritin-RBD vaccine. Three states of the ferritin–RBD nanoparticle vaccines specific to SARS-CoV-2 were modeled, including the unglycosylated, glycosylated, and modified with additional O-glycans at the ferritin–RBD interface. It was shown that glycosylation generally maintains the nanoparticle’s stability. The stability increases significantly by introducing a modified loop that includes additional glycosylation sites. If experimentally validated, these findings could be essential for improving the currently available ferritin–RBD nanoparticle vaccine and future nanoparticle vaccine designs against all sorts of Coronaviridae family.

## Data Availability

The original contributions presented in the study are included in the article/Supplementary Material; further inquiries can be directed to the corresponding author.
