# Supplementary material for: In silico design of refined ferritin-SARS-CoV-2 glyco-RBD nanoparticle vaccine
Source: Front Mol Biosci. 2022 Sep 6;9:976490. doi: 10.3389/fmolb.2022.976490 (PMC9486171; doi:10.3389/fmolb.2022.976490)

**In-silico Design of Refined Ferritin-SARS-CoV-2 RBD Nanoparticle Vaccine**

Seyedeh Zeinab Masoomi Nomandan^1^, Maryam Azimzadeh Irani^1*^ and Seyed Masoud Hosseini^1^

^1^Faculty of Life Sciences and Biotechnology, Shahid Beheshti University, Tehran, Iran.

*Corresponding author:

E-mail: m_azimzadeh@sbu.ac.ir

Address: Faculty of Life Sciences and Biotechnology, Shahid Beheshti University, Tehran, Iran. Post Code: 1983969411.

Tel: +98-21-29901

Fax: +98- 21-22431919

**Supplementary Tables**

Table S1. Protein Data Bank (PDB) spike protein structures refinement for docking.

| spike protein | | | | | | | |
| --- | --- | --- | --- | --- | --- | --- | --- |
| ID | resolution | method | weight | missing aa | up or down | year | explanation |
| [6VXX](https://www.rcsb.org/structure/6VXX) | 2.80 Å | EM | 438.26 kDa | 927 | down | 2/25/2020 | Structure of the SARS-CoV-2 spike glycoprotein. |
| [6XM4](https://www.rcsb.org/structure/6XM4) | 2.90 Å | EM | 436.03 kDa | 682 | up | 6/29/2020 | Structure of SARS-CoV-2 spike at pH 5.5, single RBD up. |
| [6XM3](https://www.rcsb.org/structure/6XM3) | 2.90 Å | EM | 435.82 kDa | 682 | up | 6/29/2020 | Structure of SARS-CoV-2 spike at pH 5.5, single RBD up. |
| [6XLU](https://www.rcsb.org/structure/6XLU) | 2.40 Å | EM | 436.30 kDa | 647 |  | 6/29/2020 | Structure of SARS-CoV-2 spike at pH 4.0. |
| [6XM5](https://www.rcsb.org/structure/6XM5) | 3.10 Å | EM | 435.40 kDa | 676 | down | 6/29/2020 | Structure of SARS-CoV-2 spike at pH 5.5, all RBDs down. |
| [6XM0](https://www.rcsb.org/structure/6XM0) | 2.70 Å | EM | 434.99 kDa | 700 |  | 6/29/2020 | Consensus structure of SARS-CoV-2 spike at pH 5.5. |
| [7JWY](https://www.rcsb.org/structure/7JWY) | 2.50 Å | EM | 436.71 kDa | 647 |  | 8/26/2020 | Structure of SARS-CoV-2 spike at pH 4.5. |
| [7KMS](https://www.rcsb.org/structure/7KMS) | 3.64 Å | EM | 650.22 kDa | 768 |  | 11/3/2020 | Cryo-EM structure of triple ACE2-bound SARS-CoV-2 trimer spike at pH 7.4. |
| [7KNE](https://www.rcsb.org/structure/7KNE) | 3.85 Å | EM | 508.63 kDa | 824 |  | 11/4/2020 | Cryo-EM structure of single ACE2-bound SARS-CoV-2 trimer spike at pH 5.5. |
| [7KNB](https://www.rcsb.org/structure/7KNB) | 3.93 Å | EM | 508.65 kDa | 824 |  | 11/4/2020 | Cryo-EM structure of single ACE2-bound SARS-CoV-2 trimer spike at pH 7.4. |
| [7KNH](https://www.rcsb.org/structure/7KNH) | 3.74 Å | EM | 577.82 kDa | 798 |  | 11/4/2020 | Cryo-EM Structure of Double ACE2-Bound SARS-CoV-2. |
| [6VYB](https://www.rcsb.org/structure/6VYB) | 3.20 Å | EM | 437.44 kDa | 968 | up | 2/25/2020 | SARS-CoV-2 spike ectodomain structure. |
| [6ZGE](https://www.rcsb.org/structure/6ZGE) | 2.60 Å | EM | 444.19 kDa | 567 | down | 6/18/2020 | Uncleavable Spike Protein of SARS-CoV-2. |
| [6ZGI](https://www.rcsb.org/structure/6ZGI) | 2.90 Å | EM | 444.00 kDa | 567 | down | 6/18/2020 | Furin Cleaved Spike Protein of SARS-CoV-2. |
| [7DF3](https://www.rcsb.org/structure/7DF3) | 2.70 Å | EM | 436.99 kDa | 519 | down | 11/6/2020 | SARS-CoV-2 S trimer. |
| [7DWZ](https://www.rcsb.org/structure/7DWZ) | 3.30 Å | EM | 442.82 kDa | 830 | up | 1/18/2021 | S protein of SARS-CoV-2 in the active conformation. |
| [7DX1](https://www.rcsb.org/structure/7DX1) | 3.10 Å | EM | 442.64 kDa | 935 |  | 1/18/2021 | S protein of SARS-CoV-2 D614G mutant. |
| [6ZB5](https://www.rcsb.org/structure/6ZB5) | 2.85 Å | EM | 425.75 kDa | 681 | down | 6/7/2020 | SARS CoV-2 Spike protein, Closed conformation, C3 symmetry. |
| [6ZB4](https://www.rcsb.org/structure/6ZB4) | 3.03 Å | EM | 426.00 kDa | 644 | down | 6/6/2020 | SARS CoV-2 Spike protein, Closed conformation, C1 symmetry. |
| [7AD1](https://www.rcsb.org/structure/7AD1) | 2.92 Å | EM | 434.10 kDa | 1061 | up | 9/14/2020 | Cryo-EM structure of a prefusion stabilized SARS-CoV-2 Spike. |
| [7DWY](https://www.rcsb.org/structure/7DWY) | 2.70 Å | EM | 446.07 kDa | 552 | down | 1/18/2021 | S protein of SARS-CoV-2 in the locked conformation. |
| [6X6P](https://www.rcsb.org/structure/6X6P) | 3.22 Å | EM | 434.87 kDa | 771 |  | 5/28/2020 | Characterization of the SARS-CoV-2 S Protein. |
| [7DDD](https://www.rcsb.org/structure/7DDD) | 3.00 Å | EM | 420.17 kDa | 519 | down | 10/28/2020 | SARS-Cov2 S protein at close state. |
| [7KDJ](https://www.rcsb.org/structure/7KDJ) | 3.49 Å | EM | 440.69 kDa | 989 |  | 10/8/2020 | SARS-CoV-2 D614G 1-RBD-up Spike Protein. |
| [7KDK](https://www.rcsb.org/structure/7KDK) | 2.80 Å | EM | 440.79 kDa | 948 |  | 10/8/2020 | SARS-CoV-2 D614G 3 RBD down Spike Protein. |
| [7KDH](https://www.rcsb.org/structure/7KDH) | 3.33 Å | EM | 440.13 kDa | 973 |  | 10/8/2020 | SARS-CoV-2 RBD up Spike Protein. |
| [7KDI](https://www.rcsb.org/structure/7KDI) | 3.26 Å | EM | 441.51 kDa | 948 |  | 10/8/2020 | SARS-CoV-2 D614G 3 RBD down Spike Protein. |
| [7KDL](https://www.rcsb.org/structure/7KDL) | 2.96 Å | EM | 439.96 kDa | 989 | up | 10/8/2020 | SARS-CoV-2 D614G 1-RBD up Spike Protein. |
| [7KDG](https://www.rcsb.org/structure/7KDG) | 3.01 Å | EM | 440.97 kDa | 948 |  | 10/8/2020 | SARS-CoV-2 RBD down Spike Protein. |
| [7KEA](https://www.rcsb.org/structure/7KEA) | 3.33 Å | EM | 439.74 kDa | 989 |  | 10/10/2020 | SARS-CoV-2 D614G 1-RBD-up Spike Protein. |
| [7KEB](https://www.rcsb.org/structure/7KEB) | 3.48 Å | EM | 439.96 kDa | 989 |  | 10/10/2020 | SARS-CoV-2 D614G 1RBD up Spike Protein. |
| [7KE9](https://www.rcsb.org/structure/7KE9) | 3.08 Å | EM | 439.96 kDa | 989 |  | 10/10/2020 | SARS-CoV-2 D614G 1-RBD-up Spike Protein. |
| [7KE4](https://www.rcsb.org/structure/7KE4) | 3.21 Å | EM | 440.79 kDa | 948 |  | 10/10/2020 | SARS-CoV-2 D614G 3 RBD down Spike. |
| [7KE7](https://www.rcsb.org/structure/7KE7) | 3.32 Å | EM | 440.79 kDa | 948 |  | 10/10/2020 | SARS-CoV-2 D614G 3-RBD-down Spike Protein. |
| [7KE8](https://www.rcsb.org/structure/7KE8) | 3.26 Å | EM | 440.79 kDa | 948 |  | 10/10/2020 | SARS-CoV-2 D614G 3 RBD down Spike Protein Trimer. |
| [7KE6](https://www.rcsb.org/structure/7KE6) | 3.10 Å | EM | 440.79 kDa | 948 | down | 10/10/2020 | SARS-CoV-2 D614G 3 RBD down Spike Protein. |
| [6X79](https://www.rcsb.org/structure/6X79) | 2.90 Å | EM | 435.73 kDa | 993 | down | 5/29/2020 | Prefusion SARS-CoV-2 S ectodomain trimer. |
| [6XKL](https://www.rcsb.org/structure/6XKL) | 3.21 Å | EM | 438.14 kDa | 944 | up | 6/26/2020 | SARS-CoV-2 HexaPro S One RBD up. |
| [7JWB](https://www.rcsb.org/structure/7JWB) | 3.20 Å | EM | 445.52 kDa | 756 |  | 8/25/2020 | SARS CoV2 Spike ectodomain. |
| [6Z97](https://www.rcsb.org/structure/6Z97) | 3.40 Å | EM | 437.84 kDa | 889 |  | 6/3/2020 | Structure of the prefusion SARS-CoV-2 spike glycoprotein. |
| [6XF5](https://www.rcsb.org/structure/6XF5) | 3.45 Å | EM | 429.23 kDa | 738 | up | 6/15/2020 | Cryo-EM structure of a biotinylated SARS-CoV-2 spike probe in the prefusion state. |
| [7NT9](https://www.rcsb.org/structure/7NT9) | 3.36 Å | EM | 446.60 kDa | 400 | down | 3/9/2021 | Trimeric SARS-CoV-2 spike ectodomain in complex with biliverdin. |
| [6ZOX](https://www.rcsb.org/structure/6ZOX) | 3.00 Å | EM | 428.75 kDa | 690 |  | 7/8/2020 | Structure of Disulphide-stabilized SARS-CoV-2 Spike Protein Trime. |
| [6ZOY](https://www.rcsb.org/structure/6ZOY) | 3.10 Å | EM | 427.91 kDa | 678 | down | 7/8/2020 | Structure of Disulphide-stabilized SARS-CoV-2 Spike Protein Trimer. |
| [6ZP1](https://www.rcsb.org/structure/6ZP1) | 3.30 Å | EM | 428.50 kDa | 690 | down | 7/8/2020 | Structure of SARS-CoV-2 Spike Protein Trimer. |
| [6ZP0](https://www.rcsb.org/structure/6ZP0) | 3.00 Å | EM | 428.00 kDa | 651 | down | 7/8/2020 | Structure of SARS-CoV-2 Spike Protein Trimer. |
| [7DX5](https://www.rcsb.org/structure/7DX5) | 3.30 Å | EM | 539.15 kDa | 1122 | up | 1/18/2021 | S protein of SARS-CoV-2 bound with PD of ACE2. |
| [7DX6](https://www.rcsb.org/structure/7DX6) | 3.00 Å | EM | 539.13 kDa | 1122 | up | 1/18/2021 | S protein of SARS-CoV-2 bound with PD of ACE2. |
| [7DX7](https://www.rcsb.org/structure/7DX7) | 3.40 Å | EM | 539.13 kDa | 1122 | up | 1/18/2021 | Trypsin-digested S protein of SARS-CoV-2 bound with PD of ACE2. |
| [7DX8](https://www.rcsb.org/structure/7DX8) | 2.90 Å | EM | 635.45 kDa | 1309 | up | 1/18/2021 | Trypsin-digested S protein of SARS-CoV-2 bound with PD of ACE2. |
| [6ZP2](https://www.rcsb.org/structure/6ZP2) | 3.10 Å | EM | 430.85 kDa | 573 | down | 7/8/2020 | Structure of SARS-CoV-2 Spike Protein Trimer. |
| [7A4N](https://www.rcsb.org/structure/7A4N) | 2.75 Å | EM | 432.23 kDa | 1002 | down | 8/20/2020 | Cryo-EM structure of a prefusion stabilized SARS-CoV-2 Spike. |
| [6X2A](https://www.rcsb.org/structure/6X2A) | 3.30 Å | EM | 422.37 kDa | 944 | up | 5/20/2020 | SARS-CoV-2 u1S2q 1-RBD Up Spike Protein Trimer |
| [6X2C](https://www.rcsb.org/structure/6X2C) | 3.20 Å | EM | 422.37 kDa | 903 | down | 5/20/2020 | SARS-CoV-2 u1S2q All Down RBD State Spike Protein Trimer |
| [6X29](https://www.rcsb.org/structure/6X29) | 2.70 Å | EM | 422.18 kDa | 903 | down | 5/20/2020 | SARS-CoV-2 rS2d Down State Spike Protein Trimer |
| [7LYN](https://www.rcsb.org/structure/7LYN) | 3.32 Å | EM | 435.38 kDa | 879 | up | 3/7/2021 | South African (B.1.351) SARS-CoV-2 spike protein. |
| [7LYO](https://www.rcsb.org/structure/7LYO) | 3.32 Å | EM | 435.82 kDa | 882 | up | 3/7/2021 | South African (B.1.351) SARS-CoV-2 spike protein. |
| [7LWW](https://www.rcsb.org/structure/7LWW) | 3.00 Å | EM | 438.07 kDa | 882 | up | 3/1/2021 | Triple mutant (K417N-E484K-N501Y) SARS-CoV-2 spike protein. |
| [7LWV](https://www.rcsb.org/structure/7LWV) | 3.12 Å | EM | 433.91 kDa | 875 | up | 3/1/2021 | UK (B.1.1.7) SARS-CoV-2 spike protein variant. |
| [7LWU](https://www.rcsb.org/structure/7LWU) | 3.22 Å | EM | 434.13 kDa | 875 | up | 3/1/2021 | UK (B.1.1.7) SARS-CoV-2 spike protein variant. |
| [7LWT](https://www.rcsb.org/structure/7LWT) | 3.19 Å | EM | 436.84 kDa | 875 | up | 3/1/2021 | UK (B.1.1.7) SARS-CoV-2 spike protein variant. |
| [7LXZ](https://www.rcsb.org/structure/7LXZ) | 2.60 Å | EM | 593.31 kDa | 684 |  | 3/5/2021 | SARS-CoV-2 S/S2M11/S2L28 Global Refinement |
| [7N1Q](https://www.rcsb.org/structure/7N1Q) | 2.90 Å | EM | 452.65 kDa | 634 |  | 5/28/2021 | Structural basis for enhanced infectivity and immune evasion of SARS-CoV-2 variants. |
| [6ZOW](https://www.rcsb.org/structure/6ZOW) | 3.00 Å | EM | 588.06 kDa | 2290 | up | 7/7/2020 | SARS-CoV-2 spike in prefusion state |
| [6ZP7](https://www.rcsb.org/structure/6ZP7) | 3.30 Å | EM | 445.67 kDa | 1003 | up | 7/8/2020 | SARS-CoV-2 spike in prefusion state. |
| [6ZP5](https://www.rcsb.org/structure/6ZP5) | 3.10 Å | EM | 445.67 kDa | 1003 | up | 7/8/2020 | SARS-CoV-2 spike in prefusion state. |
| [7KJ4](https://www.rcsb.org/structure/7KJ4) | 3.40 Å | EM | 642.23 kDa | 825 |  | 10/25/2020 | SARS-CoV-2 Spike Glycoprotein with three ACE2 Bound. |
| [7LWI](https://www.rcsb.org/structure/7LWI) | 3.07 Å | EM | 432.96 kDa | 855 | down | 3/1/2021 | Mink Cluster 5-associated SARS-CoV-2 spike protein. |
| [7LWO](https://www.rcsb.org/structure/7LWO) | 2.85 Å | EM | 434.23 kDa | 878 | up | 3/1/2021 | Mink Cluster 5-associated SARS-CoV-2 spike protein. |
| [7LWN](https://www.rcsb.org/structure/7LWN) | 2.94 Å | EM | 434.42 kDa | 878 | up | 3/1/2021 | Mink Cluster 5-associated SARS-CoV-2 spike protein. |
| [7LWQ](https://www.rcsb.org/structure/7LWQ) | 3.44 Å | EM | 432.72 kDa | 1433 |  | 3/1/2021 | \| Mink Cluster 5-associated SARS-CoV-2 spike. \| \| --- \| |
| [7LWP](https://www.rcsb.org/structure/7LWP) | 3.01 Å | EM | 433.18 kDa | 876 | up | 3/1/2021 | Mink Cluster 5-associated SARS-CoV-2 spike protein. |
| [7LWK](https://www.rcsb.org/structure/7LWK) | 2.92 Å | EM | 432.96 kDa | 852 | down | 3/1/2021 | Mink Cluster 5-associated SARS-CoV-2 spike protein. |
| [7LWJ](https://www.rcsb.org/structure/7LWJ) | 3.24 Å | EM | 432.96 kDa | 852 | down | 3/1/2021 | Mink Cluster 5-associated SARS-CoV-2 spike protein. |
| [7LWM](https://www.rcsb.org/structure/7LWM) | 2.83 Å | EM | 434.46 kDa | 878 | up | 3/1/2021 | Mink Cluster 5-associated SARS-CoV-2 spike protein. |
| [7LWL](https://www.rcsb.org/structure/7LWL) | 2.84 Å | EM | 432.96 kDa | 855 | down | 3/1/2021 | Mink Cluster 5-associated SARS-CoV-2 spike protein . |
| [7MJG](https://www.rcsb.org/structure/7MJG) | 2.81 Å | EM | 440.38 kDa | 744 |  | 4/20/2021 | Cryo-EM structure of the SARS-CoV-2 N501Y mutant spike protein ectodomain. |
| [7LWS](https://www.rcsb.org/structure/7LWS) | 3.22 Å | EM | 440.00 kDa | 855 | down | 3/1/2021 | UK (B.1.1.7) SARS-CoV-2 S-GSAS-D614G variant spike protein. |
| [6XRA](https://www.rcsb.org/structure/6XRA) | 3.00 Å | EM | 446.08 kDa | 2886 |  | 7/11/2020 | Distinct conformational states of SARS-CoV-2 spike protein. |
| [6XR8](https://www.rcsb.org/structure/6XR8) | 2.90 Å | EM | 459.49 kDa | 609 |  | 7/11/2020 | Distinct conformational states of SARS-CoV-2 spike protein. |
| [7N1T](https://www.rcsb.org/structure/7N1T) | 3.11 Å | EM | 456.09 kDa | 570 |  | 5/28/2021 | Structural basis for enhanced infectivity and immune evasion of SARS-CoV-2 variants. |
| [7N1U](https://www.rcsb.org/structure/7N1U) | 3.14 Å | EM | 453.83 kDa | 690 |  | 5/28/2021 | Structural basis for enhanced infectivity and immune evasion of SARS-CoV-2 variants. |
| [7N1V](https://www.rcsb.org/structure/7N1V) | 3.21 Å | EM | 453.02 kDa | 641 |  | 5/28/2021 | Structural basis for enhanced infectivity and immune evasion of SARS-CoV-2 variants. |
| [7N1W](https://www.rcsb.org/structure/7N1W) | 3.33 Å | EM | 453.02 kDa | 658 |  | 5/28/2021 | Structural basis for enhanced infectivity and immune evasion of SARS-CoV-2 variants. |
| [7EB5](https://www.rcsb.org/structure/7EB5) | 3.40 Å | EM | 442.42 kDa | 791 | up | 3/8/2021 | Cryo-EM structure of SARS-CoV-2 Spike D614G variant, two RBD-up. |
| [7DX2](https://www.rcsb.org/structure/7DX2) | 3.30 Å | EM | 442.64 kDa | 935 |  | 1/18/2021 | Trypsin-digested S protein of SARS-CoV-2 D614G mutant. |
| [7DX0](https://www.rcsb.org/structure/7DX0) | 3.20 Å | EM | 442.82 kDa | 935 |  | 1/18/2021 | Trypsin-digested S protein of SARS-CoV-2. |
| [7KRQ](https://www.rcsb.org/structure/7KRQ) | 3.40 Å | EM | 457.73 kDa | 567 |  | 11/20/2020 | Structural impact on SARS-CoV-2 spike protein by D614G substitution. |
| [7KRS](https://www.rcsb.org/structure/7KRS) | 3.20 Å | EM | 455.42 kDa | 614 |  | 11/20/2020 | Structural impact on SARS-CoV-2 spike protein by D614G substitution. |
| [6VSB](https://www.rcsb.org/structure/6VSB) | 3.46 Å | EM | 440.39 kDa | 959 | up | 2/10/2020 | Prefusion 2019-nCoV spike glycoprotein. |
| [7E7D](https://www.rcsb.org/structure/7E7D) | 3.20 Å | EM | 523.12 kDa | 1320 |  | 2/25/2021 | Cryo-EM structure of the SARS-CoV-2 wild-type S-Trimer. |
| [7E7B](https://www.rcsb.org/structure/7E7B) | 2.60 Å | EM | 526.77 kDa | 1254 |  | 2/25/2021 | Cryo-EM structure of the SARS-CoV-2 furin site. |

Table S2. protein data bank spike protein S1 in complex with ACE2 structures refinement for docking.

| **Spike protein S1 in complex with ACE2** | | | | | | | |
| --- | --- | --- | --- | --- | --- | --- | --- |
| [6VW1](https://www.rcsb.org/structure/6VW1) | 2.68 Å | X-Ray | 192.75 kDa | 49 | up | 2/18/2020 | Structure of SARS-CoV-2 chimeric receptor-binding domain. |
| [6M0J](https://www.rcsb.org/structure/6M0J) | 2.45 Å | X-Ray | 97.14 kDa | 41 | up | 2/21/2020 | Crystal structure of SARS-CoV-2 spike receptor-binding domain bound with ACE2. |
| [7C8D](https://www.rcsb.org/structure/7C8D) | 3.00 Å | EM | 107.07 kDa | 135 | up | 5/29/2020 | Cryo-EM structure of cat ACE2 and SARS-CoV-2 RBD. |
| [7DMU](https://www.rcsb.org/structure/7DMU) | 3.20 Å | X-Ray | 196.43 kDa | 62 | up | 12/7/2020 | Structure of SARS-CoV-2 spike receptor-binding domain complexed. |
| [6LZG](https://www.rcsb.org/structure/6LZG) | 2.50 Å | X-Ray | 93.50 kDa | 14 | up | 2/19/2020 | Structure of novel coronavirus spike receptor-binding domain complexed. |
| [7NXC](https://www.rcsb.org/structure/7NXC) | 3.14 Å | X-Ray | 94.42 kDa | 18 | up | 3/17/2021 | Crystal structure of the receptor binding domain of SARS-CoV-2. |
| [7BH9](https://www.rcsb.org/structure/7BH9) | 2.90 Å | EM | 94.25 kDa | 82 | up | 1/11/2021 | SARS-CoV-2 RBD-62 in complex with ACE2 peptidase domain. |
| [7DDO](https://www.rcsb.org/structure/7DDO) | 3.40 Å | EM | 93.59 kDa | 15 | up | 10/29/2020 | Cryo-EM structure of human ACE2 and GD/1/2019 RBD. |
| [7DDP](https://www.rcsb.org/structure/7DDP) | 3.40 Å | EM | 93.73 kDa | 15 | up | 10/29/2020 | Cryo-EM structure of human ACE2 and GX/P2V/2017 RBD. |
| [7DMU](https://www.rcsb.org/structure/7DMU) | 3.20 Å | X-Ray | 196.43 kDa | 62 | up | 12/7/2020 | Structure of SARS-CoV-2 spike receptor-binding domain. |
| [7LO4](https://www.rcsb.org/structure/7LO4) | 2.46 Å | X-Ray | 93.31 kDa | 0 | up | 2/9/2021 | SARS-CoV-2 spike receptor-binding domain. |

Table S3. protein data bank ferritin structures refinement for docking.

| **ferritin** | | | | | | | |
| --- | --- | --- | --- | --- | --- | --- | --- |
| [2FG8](https://www.rcsb.org/structure/2FG8) | 2.50 Å | X-Ray | 160.67 kDa | 0 |  | 12/21/2005 | Structure of Human Ferritin L Chain. |
| [2FHA](https://www.rcsb.org/structure/2FHA) | 1.90 Å | X-Ray | 21.33 kDa | 11 |  | 3/3/1997 | Human h chain ferritin. |
| [2FFX](https://www.rcsb.org/structure/2FFX) | 1.90 Å | X-Ray | 21.47 kDa | 0 |  | 12/20/2005 | Structure of Human Ferritin L. Chain. |
| [2FG4](https://www.rcsb.org/structure/2FG4) | 2.10 Å | X-Ray | 21.38 kDa | 3 |  | 12/21/2005 | Structure of Human Ferritin L Chain. |
| [3AJO](https://www.rcsb.org/structure/3AJO) | 1.52 Å | X-Ray | 21.42 kDa | 10 |  | 6/11/2010 | Crystal structure of wild-type human ferritin H chain. |
| [6KE2](https://www.rcsb.org/structure/6KE2) | 1.80 Å | X-Ray | 21.37 kDa | 12 |  | 7/3/2019 | ABloop reengineered Ferritin Nanocage. |
| [4DYZ](https://www.rcsb.org/structure/4DYZ) | 2.30 Å | X-Ray | 20.27 kDa | 0 |  | 2/29/2012 | Crystal Structure of the apo form of Human H-Ferritin. |
| [5N27](https://www.rcsb.org/structure/5N27) | 1.74 Å | X-Ray | 21.53 kDa | 8 |  | 2/7/2017 | X-ray structure of human heavy chain ferritin. |
| [6B8G](https://www.rcsb.org/structure/6B8G) | 1.13 Å | X-Ray | 21.64 kDa | 10 |  | 10/7/2017 | Twice-Contracted Human Heavy-Chain Ferritin. |
| [6B8F](https://www.rcsb.org/structure/6B8F) | 1.06 Å | X-Ray | 21.64 kDa | 10 |  | 10/7/2017 | Contracted Human Heavy-Chain Ferritin. |

**Supplementary Figures**

Figure S1. Residue cross correlation matrix of RBDfN is calculated over 100ns MD simulations for CA atoms. Shades of red and blue spots present atoms correlated and anti-correlated motions. The amino acid residues of RBD and ferritin are marked on the x and y axes with green and yellow, respectively. The values of the correlations range from -1 to 1 as shown in the bar on the right.


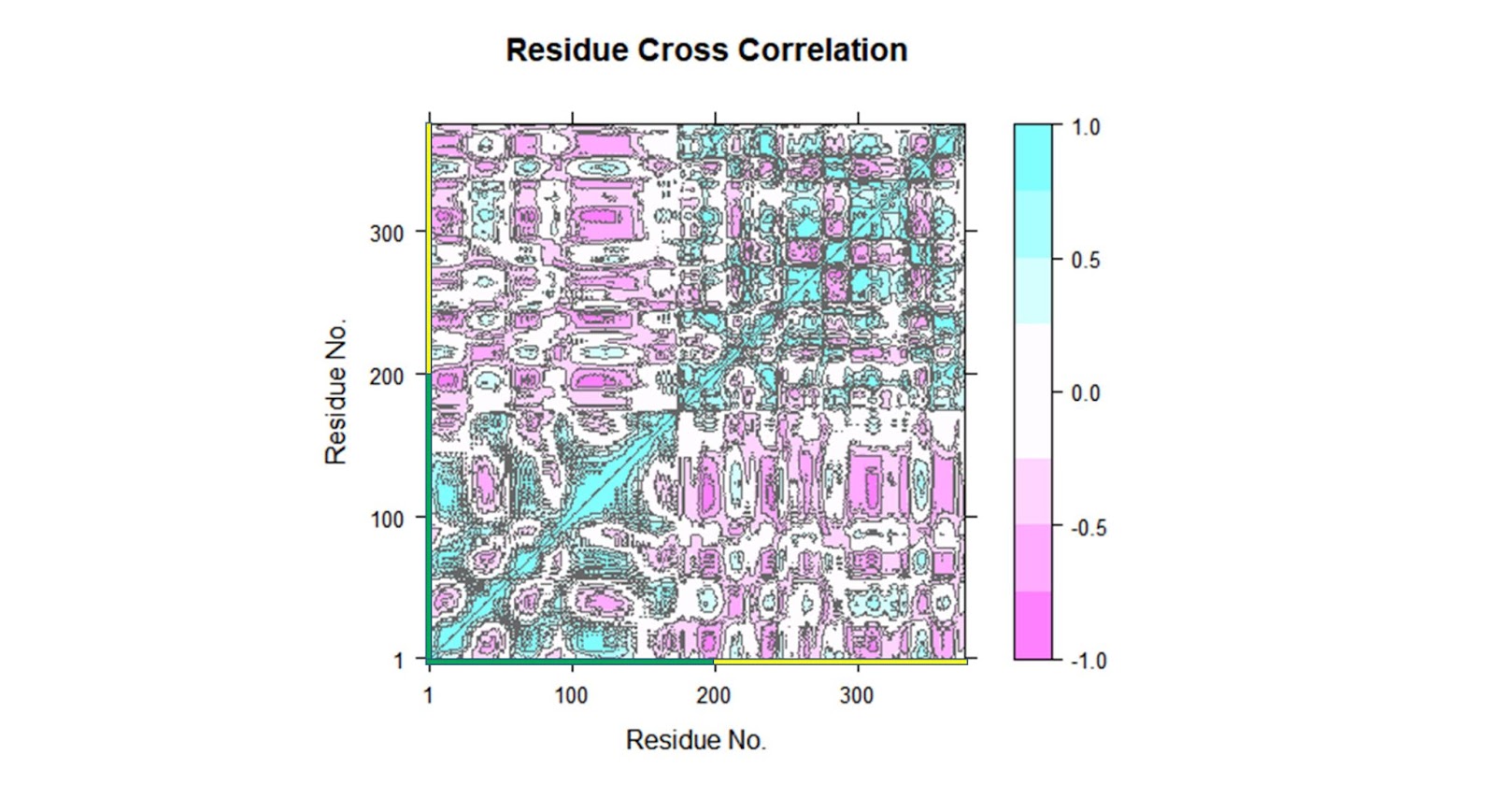

Supplement: Supplementary file 1 [file DataSheet1.DOCX]
